# Transglutaminase 2 regulates osteoclast differentiation via a Blimp1-dependent pathway

**DOI:** 10.1038/s41598-017-11246-5

**Published:** 2017-09-06

**Authors:** Woo-Shin Kim, Haemin Kim, Eui Man Jeong, Hyung Joon Kim, Zang Hee Lee, In-Gyu Kim, Hong-Hee Kim

**Affiliations:** 1 Department of Cell and Developmental Biology, BK21 Program and Dental Research Institute, Seoul National University, Seoul, 03080 Korea; 2 Department of Biochemistry and Molecular Biology, Seoul National University College of Medicine, Seoul, 110-799 Korea; 3 Department of Oral Physiology, School of Dentistry, Pusan National University, Yangsan, 50612 Korea

## Abstract

Transglutaminase 2 (TG2) performs multiple reactions, including transamidation, and also plays a role in signal transduction as a GTP-binding protein. In this study, we reveal that TG2 controls osteoclast differentiation and bone homeostasis in mice. Osteoclasts specifically expressed the TG2 isoform among eight TG family members. Suppression in TG2 expression with siRNA led to increased osteoclast formation from primary mouse precursor cells in response to receptor activator of nuclear factor kappaB ligand (RANKL). This osteoclastogenic effect of TG2 knockdown was associated with enhanced induction of c-Fos and NFATc1 by RANKL. Moreover, TG2 knockdown up-regulated B lymphocyte-induced maturation protein 1 (Blimp1), which represses anti-osteoclastogenic genes, in a manner dependent on the NF-κB signaling pathway. To the contrary, TG2 overexpression inhibited osteoclast formation and the expression of osteoclastogenic genes. Consistent with these *in vitro* results, TG2 knockout mice exhibited lower trabecular bone mass and increased number of osteoclasts compared with wild-type mice. Taken together, our results provide strong evidence that TG2 plays an important role in bone metabolism by suppressing excessive osteoclastogenesis via the regulation of the NF-κB-Blimp1 signaling pathway.

## Introduction

Bone is a dynamic tissue that is continuously remodeled through the coupled actions of osteoclasts and osteoblasts^1^. The balance of the bone resorption activity of osteoclasts and bone formation by osteoblasts is critical to the maintenance of bone homeostasis. Inadequate bone remodeling accounts for the pathologic skeletal states associated with rheumatoid arthritis, periodontitis, and Paget’s disease, as well as osteoporosis^2^. Osteoclasts are generated by differentiation of the monocyte/macrophage lineage of hematopoietic cells^3^. Two factors, macrophage-colony stimulating factor (M-CSF) and receptor activator of nuclear factor κB (NF-κB) ligand (RANKL), play fundamental roles in osteoclast differentiation. M-CSF is critical for the commitment, proliferation, and survival of monocyte/macrophage lineage cells, while RANKL induces the differentiation and fusion of precursor cells into multinucleated cells (MNCs) expressing osteoclast specific genes, such as tartrate-resistant acid phosphatase (TRAP)^4, 5^.

RANKL binding to its receptor, RANK, on osteoclast precursor cells stimulates signaling cascades resulting in activation of the mitogen-activated protein kinases (MAPKs), including extracellular signal-regulated kinase (ERK), c-Jun N-terminal kinase (JNK), and p38^6^. RANKL stimulation also leads to the activation and induction of the transcription factors NF-κB, c-Fos, and nuclear factor of activated T-cells cytoplasmic 1 (NFATc1)^7–9^. These transcription factors promote the expression of osteoclast marker genes, including TRAP, v-ATPase subunit d2 (ATP6v0d2), and dendritic cell-specific transmembrane protein (DC-STAMP)^10, 11^. Mature osteoclasts form a ring-shaped sealing zone of tight contact with the bone surface surrounding the resorption lacuna^12^. These polarized osteoclasts dissolve the bone matrix by secreting acid and proteases, such as TRAP, cathepsin K, and matrix metalloprotease 9 (MMP9), into the resorption pit.

B lymphocyte-induced maturation protein 1 (Blimp1) has been characterized as a transcriptional repressor involved in the differentiation and/or functioning of macrophages and lymphocytes^13^. Recently, Blimp1 was also reported to regulate RANKL-mediated osteoclast differentiation by suppressing interferon regulatory factor-8 (IRF-8) and v-maf musculoaponeurotic fibrosarcoma oncogene family protein B (MafB)^14^. As IRF-8 and MafB have been shown to reduce the expression and function of NFATc1^15, 16^, Blimp1 might ensure the maintenance of NFATc1 activity during osteoclastogenesis by repressing IRF-8 and MafB.

The transglutaminase (TG) family consists of eight distinct members: factor XIII A (FXIIIA), TG1 (or keratinocyte TG), TG2 (or tissue TG), TG3 (or epidermal TG), TG4 (or prostate TG), TG5 (or TG X), TG6 (or TG Y), and TG7 (or TG Z)^17^. TG family enzymes catalyze posttranslational modifications of various substrates via transamidation, esterification, and hydrolysis reactions in a Ca^2+^-dependent manner. These TG-mediated reactions have an impact on diverse cellular responses, including proliferation^18^, differentiation^19^, death^20^, and migration^21^. Consequently, TGs modulate multiple biological processes, including development, tissue remodeling, inflammation, and wound healing^22–24^. The TG2 isoform is distinguished from other members of TG family by several unique characteristics, such as a ubiquitous expression pattern, GTP-binding and -hydrolysis, and cell-matrix interaction regulation^25^. The functions of TG2 depend on its subcellular localization and presence of its regulators. Ca^2+^ and GTP are the most important regulators of TG2 and act as switches between the two distinct functional entities of TG2, transglutaminase and GTP hydrolase, via allosteric modulation^23, 26, 27^.

Some studies have reported association of TG family members, especially TG2 and FXIIIA, with bone development and matrix mineralization^28^. TG2 and FXIIIA were shown to be expressed in hypertrophic chondrocytes and osteoblasts^28, 29^. In preosteoblastic MC3T3-E1 cells, a high level of FXIIIA activity was detected during osteoblastic differentiation and inhibition of TG activity suppressed mineralization^30^. These and other observations have implicated TG2 and FXIIIA in matrix crosslinking and assembly, the essential processes of bone mineralization^28^. However, the relationship between TG2 and osteoclasts has not been investigated. To gain a better understanding on the role of TG2 in the regulation of bone homeostasis, we examined whether TG2 is involved in the differentiation of osteoclasts using loss- and gain-of-function approaches.

## Results

### TG2 is involved in the regulation of RANKL-induced osteoclast differentiation

**Figure 1:**
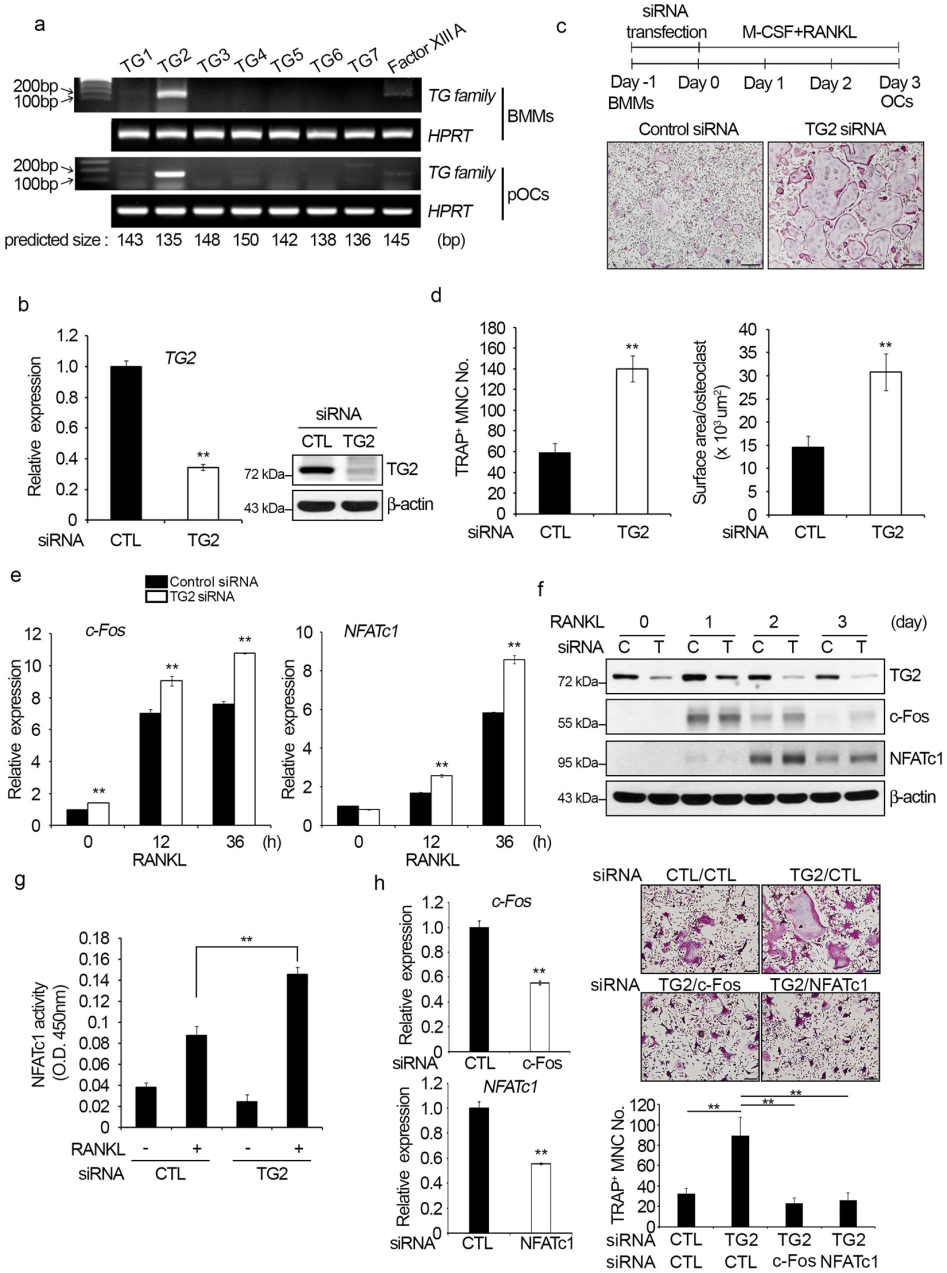
TG2 regulates RANKL-induced osteoclast differentiation. (**a**) BMMs were cultured for two days with M-CSF (30 ng/ml) alone or with M-CSF plus RANKL (100 ng/ml) to obtain pOCs. The mRNA expression levels of *TG1-TG7* and *FXIIIA* were analyzed by RT-PCR. (**b**) BMMs were transfected with control or TG2 siRNA for 18 h. The efficiency of knockdown was determined by real-time PCR and Western blotting. TG2 mRNA expression was normalized using the *HPRT* housekeeping gene, and values indicating the fold-change from control are shown. β-actin was used as a protein loading control. CTL, control. (**c**) BMMs transfected with control or TG2 siRNA were cultured with M-CSF plus RANKL for three days. Cells were stained for TRAP activity. Scale bar, 200 μm. (**d**) TRAP-positive MNCs (≥3 nuclei) were counted as osteoclasts. The surface area per osteoclast was measured using the Osteomeasure program. (**e**) The mRNA levels of *c-Fos*, and *NFATc1* were measured by real-time PCR. (**f**) Whole cell lysates were subjected to Western blot analysis with anti-TG2, -c-Fos, and -NFATc1 antibodies. C, control siRNA; T, TG2 siRNA. (**g**) Nuclear fractions were obtained from cells cultured for two days and subjected to an NFATc1 transcriptional activity assay. CTL, control. (**h**) BMMs transfected with control or c-Fos or NFATc1 siRNA were subjected to real-time PCR analyses, and values indicating the fold-change from control are shown (left). BMMs transfected with indicated combination of siRNAs were cultured with M-CSF plus RANKL for four days. Cells were stained for TRAP activity (right upper). Scale bar, 100 μm. TRAP-positive MNCs (≥3 nuclei) were counted as osteoclasts (right lower). **P < 0.005 versus control siRNA or between indicated groups. Full length gels and Western blots are presented in Supplementary Figure 9.

We first examined the expression profile of TG family members in osteoclast precursors (BMMs) and pre-fusion osteoclasts (pOCs; BMMs treated with RANKL and M-CSF for two days). Among the TG family members, *TG2* mRNA was detected at a high level in both BMMs and pOCs (Fig. 1a). *FXIIIA* mRNA was only weakly expressed, while the other TG isoforms were not detected. This expression pattern of *TG* genes was maintained during differentiation. As TG2 was the only TG members expressed at a significant level, we next investigated the role of TG2 in osteoclastogenesis by evaluating the effect of TG2 reduction using a siRNA-mediated gene knockdown system. Two different oligonucleotides, targeting different regions of TG2 mRNA, were tested. A greater than 50% reduction in TG2 expression was achieved in BMMs transfected with TG2 siRNA #1 or #2 compared to BMMs transfected with control siRNA (Fig. 1b and Supplementary Figure 1a). To test the effect of TG2 knockdown on osteoclast formation, the siRNA-transfected BMMs were incubated with RANKL and M-CSF for three days and then stained for TRAP activity. As shown in Fig. 1c and Supplementary Figure 1b, the generation of osteoclasts (TRAP-positive MNCs) was facilitated by TG2 knockdown. The surface area per osteoclast and the number of osteoclasts generated were increased in TG2-knockdown samples (Fig. 1d and Supplementary Figure 1c), indicating more number of large osteoclasts were formed. For the rest of the study TG2 siRNA #1 (designated as TG2 siRNA) was used. Similar results were obtained with the macrophage cell line RAW264.7, which has been used as an osteoclast precursor cell in many studies (Supplementary Figure 2). Moreover, TG2 knockdown in BMMs committed to osteoclastic lineage by priming with RANKL for one or two days also accelerated mature osteoclast formation (Supplementary Figure 3). As the c-Fos and NFATc1 transcription factors are essential for osteoclastogenesis and are induced and activated by RANKL^31^, we next investigated whether TG2 knockdown altered RANKL induction of c-Fos and NFATc1. The mRNA levels of *c-Fos* and *NFATc1* were significantly higher in TG2-knockdown cells than in control cells (Fig. 1e). In addition, the protein levels of the c-Fos and NFATc1 were increased more upon RANKL stimulation in TG2-knockdown cells (Fig. 1f). Consistently, a significantly higher level of NFATc1 transcriptional activity was observed in pOCs generated from TG2-knockdown cells compared with the control cells (Fig. 1g). Finally, when knockdown of c-Fos or NFATc1 was simultaneously carried out with TG2 knockdown, the enhancement of osteoclastogenesis by TG2 down-regulation was abrogated (Fig. 1h). These results suggest that, among TG family members, TG2 has a specific role in regulating osteoclast differentiation.

### Actin ring formation and bone resorption activity are increased by TG2 knockdown

**Figure 2:**
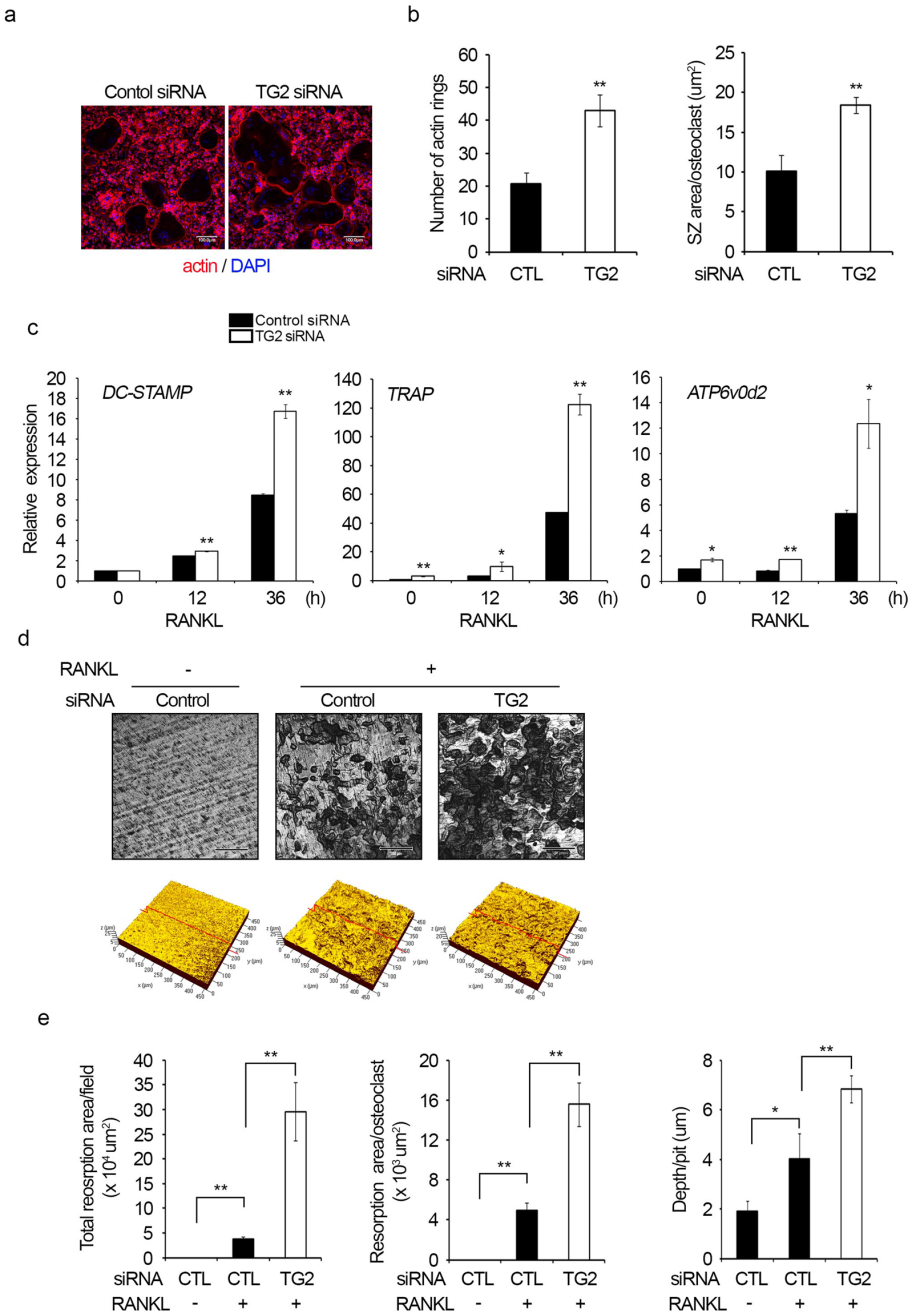
Actin ring formation and bone resorption are enhanced by TG2 knockdown. (**a**,**b**) BMMs were transfected with control or TG2 siRNA and cultured with M-CSF (30 ng/ml) and RANKL (100 ng/ml) for three days. Cells were stained with rhodamine-phalloidin conjugate and DAPI to visualize F-actin and nuclei, respectively. The number of actin rings was counted, and the sealing zone (SZ) area per osteoclast was measured. **P < 0.005 versus control siRNA. Scale bar, 100 μm. (**c**) After transfection with control or TG2 siRNA, BMMs were incubated with M-CSF (30 ng/ml) and RANKL (100 ng/ml) for the indicated number of days. The mRNA levels of *DC-STAMP*, *TRAP* and *ATP6v0d2* were measured by real-time PCR. (**d**,**e**) BMMs were transfected with control or TG2 siRNA, and a dentine resorption assay was performed. Black areas indicate resorbed lacunae on the dentin slices. The total resorption area, resorption area per osteoclast, and depth per pit were calculated from four randomly selected images. CTL, control. *P < 0.05; **P < 0.005 between indicated groups. Scale bar, 100 μm.

As TG2 appears to be involved in the regulation of osteoclast differentiation, we next examined whether the activity of osteoclast could be modulated by TG2. The sealing zone is a ring-shaped cytoskeletal structure composed of highly polymerized actin, which is crucial for tight adhesion to the bone surface and the bone-resorbing activity of osteoclasts^12, 32^. Therefore, we investigated the effect of TG2 knockdown on actin ring formation and bone resorption. The number of actin ring-positive osteoclasts generated from TG2-knockdown BMMs was higher than that from the control siRNA-transfected BMMs (Fig. 2a and b). The sealing zone area per osteoclast was also significantly increased by TG2 knockdown (Fig. 2b). As TG2 knockdown increased osteoclast size and sealing zone area, we next examined the effects of TG2 deficiency on the expression levels of genes associated with osteoclast fusion and bone resorption activity, such as *DC-STAMP*, *TRAP* and *ATP6v0d2*. As shown in Fig. 2c, the mRNA levels of *DC-STAMP*, *TRAP* and *ATP6v0d2* were higher in TG2-knockdown cells. Next, bone resorption activity was assessed using dentine slices. Consistent with the increase in the number of osteoclasts generated, TG2 knockdown caused an increase in the total area of the resorption pits formed (Fig. 2d and e). Moreover, both the resorption area per osteoclast and the depth of the pits were increased by TG2 knockdown (Fig. 2e). These data suggest that TG2 knockdown enhances actin ring formation and the resorption activity of osteoclasts, in addition to the differentiation of osteoclasts.

### Blimp1 is up-regulated by TG2 knockdown via the NF-κB signaling pathway

**Figure 3:**
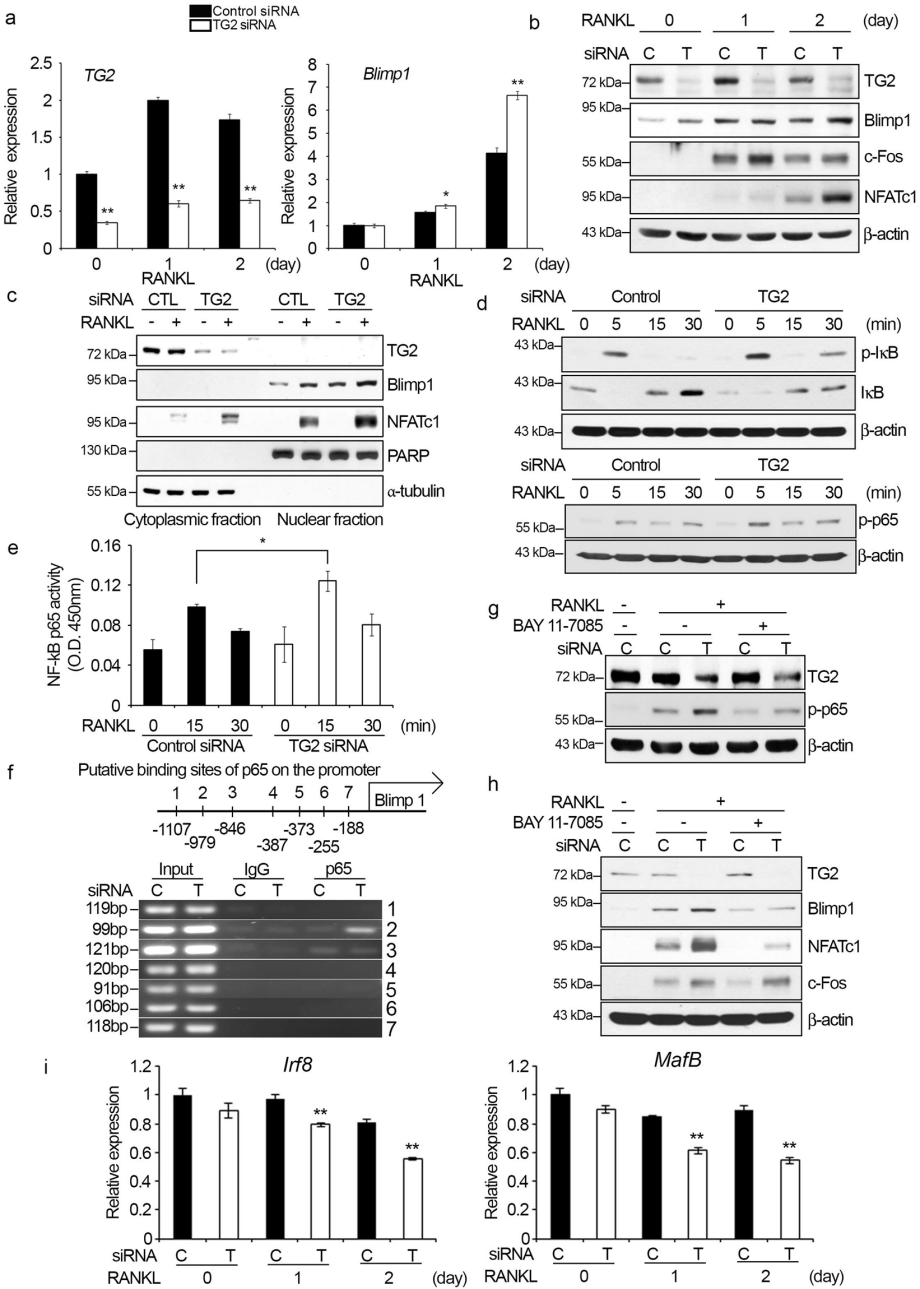
TG2 knockdown increases Blimp1 expression through the activation of the NF-κB signaling pathway. BMMs were transfected with control or TG2 siRNA and cultured for the indicated number of days in the presence of M-CSF (30 ng/ml) and RANKL (100 ng/ml). (**a**) The mRNA levels of *TG2* and *Blimp1* were assessed by real-time PCR. *P < 0.05; **P < 0.005 versus control siRNA. (**b**) The protein levels of TG2, Blimp1, c-Fos, and NFATc1 were analyzed by Western blotting. C, control siRNA; T, TG2 siRNA. (**c**) Cytoplasmic and nuclear fractions were obtained from cells cultured for two days with M-CSF and RANKL. Western blotting was performed to detect Blimp1 and NFATc1. PARP (full length) and α-tubulin were used as loading controls for cytoplasmic and nuclear extracts, respectively. CTL, control. (**d**) Cells cultured for two days after siRNA transfection were deprived of serum and factors for 5 h. Cells were then re-stimulated with RANKL (500 ng/ml) for the indicated times. Whole cell lysates were subjected to Western blot analysis with anti-phospho-IκB, -IκB and anti-phospho-p65 antibodies. (**e**) Nuclear fractions were prepared from cells stimulated as in (**d**). A p65 transcription activity assay was performed as described in Materials and methods. *P < 0.05 between indicated groups. (**f**) Seven predicted NF-κB binding sites on the promoter of Blimp1 are shown (upper). Chromatin immunoprecipitation assay with control IgG or p65 antibody was performed with RAW264.7 cells transfected with control or TG2 siRNA. PCR was performed using primer sets to detect the seven predicted sites (lower). C, control siRNA; T, TG2 siRNA. (**g**) BMMs transfected with control or TG2 siRNA were cultured with or without BAY 11-7085 (5 μM) for two days in the presence of M-CSF (30 ng/ml) and RANKL (100 ng/ml). Cells were then serum-starved for 5 h and re-stimulated with RANKL (500 ng/ml) for 5 min. Cell lysates were prepared and subjected to Western blotting. C, control siRNA; T, TG2 siRNA. (**h**) siRNA-transfected BMMs were cultured with or without BAY 11-7085 (5 μM) for two days in the presence of M-CSF (30 ng/ml) and RANKL (100 ng/ml). Western blotting was performed to assess the levels of the indicated proteins. C, control siRNA; T, TG2 siRNA. (**i**) siRNA-transfected BMMs were cultured in the presence of M-CSF (30 ng/ml) and RANKL (100 ng/ml). The mRNA levels of *Irf8* and *MafB* were assessed by real-time PCR. **P < 0.005 versus control siRNA. C, control siRNA; T, TG2 siRNA. Full length Western blots are presented in Supplementary Figure 9.

A previous study showed that TG2 had a suppressive effect on the expression of Blimp1 transcription repressor in lymphocytes^33^. Therefore, we explored the possibility that Blimp1 is involved in TG2-mediated regulation of osteoclastogenesis. We first examined whether Blimp1 expression was affected by TG2 knockdown in osteoclastogenic culture conditions. The mRNA level of *Blimp1* was increased by RANKL treatment, and this induction of *Blimp1* was significantly greater in TG2-knockdown cells than in control cells (Fig. 3a). TG2 knockdown also enhanced the RANKL induction of Blimp1 at the protein level (Fig. 3b). Next, we investigated the effect of TG2 knockdown on intranuclear level of Blimp1. Analyses of cytoplasmic and nuclear fractions revealed an increase in nuclear Blimp1 upon TG2 knockdown (Fig. 3c). Blimp1 protein was not readily detected in the cytoplasmic fraction. TG2 knockdown also increased the amount of NFATc1 protein in the nuclear fraction upon RANKL stimulation (Fig. 3c).

Because Blimp1 has been reported to have a p65 binding site in its promoter region and has been shown to be up-regulated by activation of the NF-κB signaling pathway in B cells^34^, we examined whether the increase in Blimp1 level resulting from TG2 knockdown was due to activation of the NF-κB pathway. To this end, we compared RANKL stimulation of NF-κB in control and TG2-knockdown cells. RANKL stimulation induced phosphorylation of IκB within 5 min, and the level of RANKL-induced IκB phosphorylation was further enhanced by TG2 knockdown at 5 and 30 min (Fig. 3d upper). Consistently, the total IκB level was lower in TG2-knockdown cells. Moreover, phosphorylation of p65 was increased by TG2 knockdown (Fig. 3d lower). We also examined whether p65 transcriptional activity was increased by TG2 knockdown. TG2 knockdown enhanced the activity of p65 15 min after RANKL stimulation (Fig. 3e). In contrast, the RANKL stimulation of MAPK pathways was not affected by TG2 knockdown (Supplementary Figure 4). Since multiple putative binding sites of p65 were predicted to be present on the promoter region of Blimp1, we performed the chromatin immunoprecipitation assay to assess legitimate binding of p65 regulated by TG2 knockdown. Indeed, p65 bound to predicted sites 2 and 3 of Blimp1 promoter and the enrichment by TG2 knockdown was observed at the site 2 (Fig. 3f). In addition, the NF-κB inhibitor BAY 11-7085 attenuated the increase in p65 phosphorylation by TG2 knockdown (Fig. 3g). BAY 11-7085 also significantly mitigated the effect of TG2 knockdown on the induction of Blimp1 and NFATc1 (Fig. 3h). Interestingly, the expression level of c-Fos was not affected by BAY 11-7085 treatment (Fig. 3h). These data suggest that TG2 knockdown up-regulates expression of Blimp1 and NFATc1 through the NF-κB pathway during osteoclast differentiation. We next examined the effect of TG2 knockdown on the expression of IRF-8 and MafB, transcription repressors suggested to be down-regulated by Blimp1 and suppress NFATc1 expression^14–16^. As shown in Fig. 3i, TG2 knockdown reduced the expression of MafB and IRF8 in RANKL-treated BMMs (Fig. 3i). Therefore, the regulation of NFATc1 by TG2 may involve the Blimp1-IRF8/MafB pathway in osteoclasts.

### Blimp1 mediates the effect of TG2 knockdown on osteoclastogenesis

**Figure 4:**
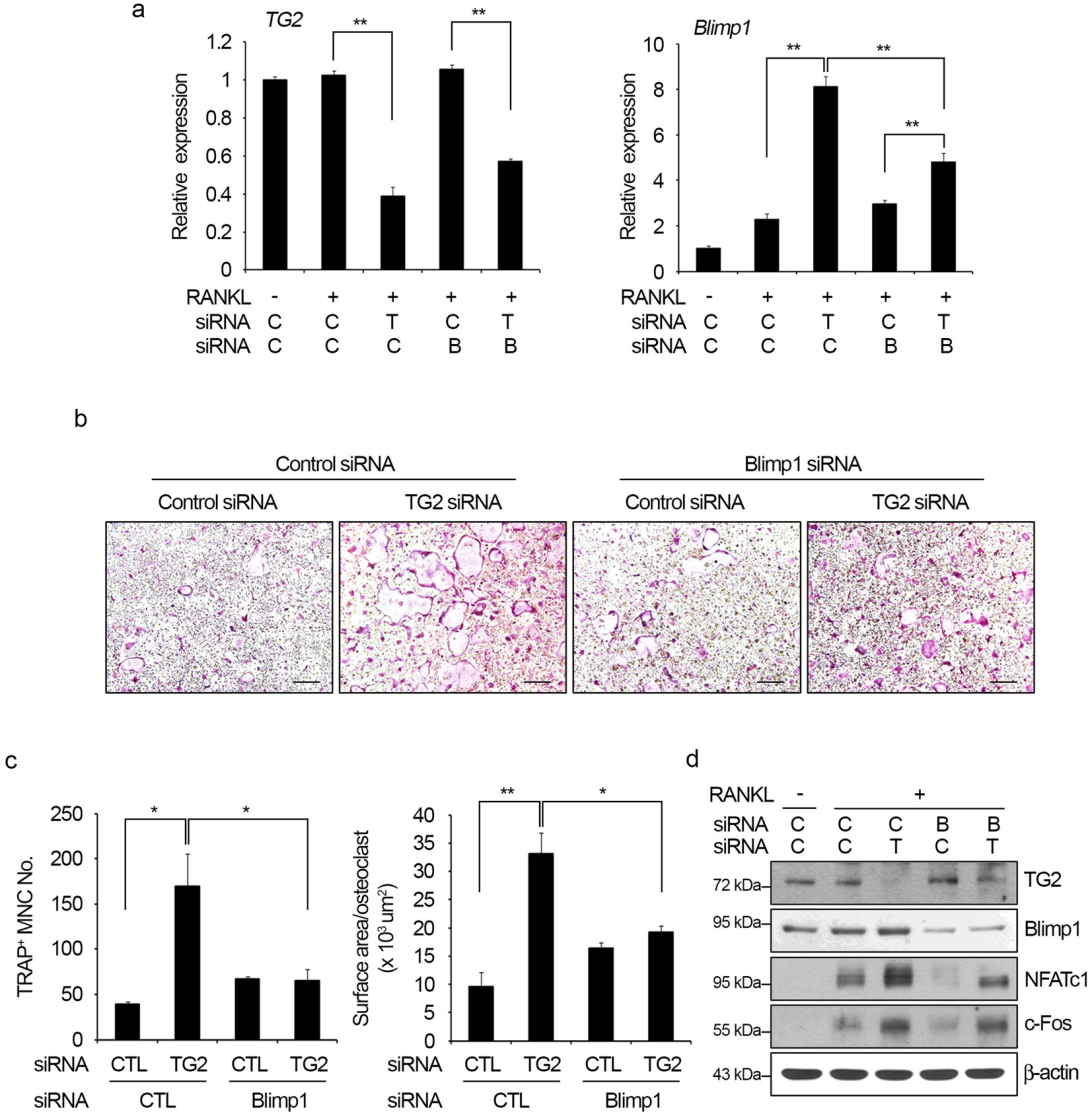
Blimp1 mediates the stimulatory effects of TG2 knockdown on osteoclastogenesis. (**a**) BMMs were transfected with TG2 and/or Blimp1 siRNA and cultured for two days with M-CSF (30 ng/ml) and RANKL (100 ng/ml). The mRNA levels of *TG2* and *Blimp1* were analyzed by real-time PCR. **P < 0.005 between indicated groups. (**b**,**c**) siRNA-transfected cells were cultured for three days with M-CSF and RANKL. The cultured cells were fixed and subjected to TRAP staining. Scale bar, 100 μm (**b**). TRAP-positive MNCs were counted, and the surface area per osteoclast was measured. *P < 0.05; **P < 0.005 between indicated groups (**c**). CTL, control. (**d**) siRNA-transfected cells were cultured for two days with M-CSF and RANKL. Cell lysates were prepared and subjected to Western blotting. C, control siRNA; T, TG2 siRNA; B, Blimp1 siRNA. Full length Western blots are presented in Supplementary Figure 10.

To further clarify the role of the TG2-NF-κB-Blimp1 axis in RANKL-induced osteoclastogenesis, we investigated the effect of TG2 and Blimp1 double-knockdown on osteoclast formation. Significant reduction in both *TG2* and *Blimp1* expression upon dual transfection of siRNAs was verified by real-time PCR (Fig. 4a). As shown earlier in Fig. 1c, TG2 knockdown significantly enhanced the formation of TRAP-positive MNCs by RANKL. This enhancement of osteoclast formation by TG2 knockdown was clearly attenuated by the addition of Blimp1 knockdown (Fig. 4b). Both the number of TRAP-positive MNCs and the surface area of osteoclasts were decreased by knockdown of Blimp1 (Fig. 4c). We also examined whether knockdown of TG2 and Blimp1 affected the expression of c-Fos and NFATc1. As previously shown in Figs 1 and 3, TG2 knockdown increased the expression level of NFATc1. Notably, Blimp1 knockdown prominently suppressed the up-regulation of NFATc1 resulting from TG2 knockdown (Fig. 4d). In contrast, the expression level of c-Fos was only slightly affected by Blimp1 knockdown (Fig. 4d). These results indicate that Blimp1 mediates the increases of osteoclast formation and NFATc1 induction by TG2 knockdown.

### TG2 overexpression suppresses RANKL-induced osteoclast differentiation

**Figure 5:**
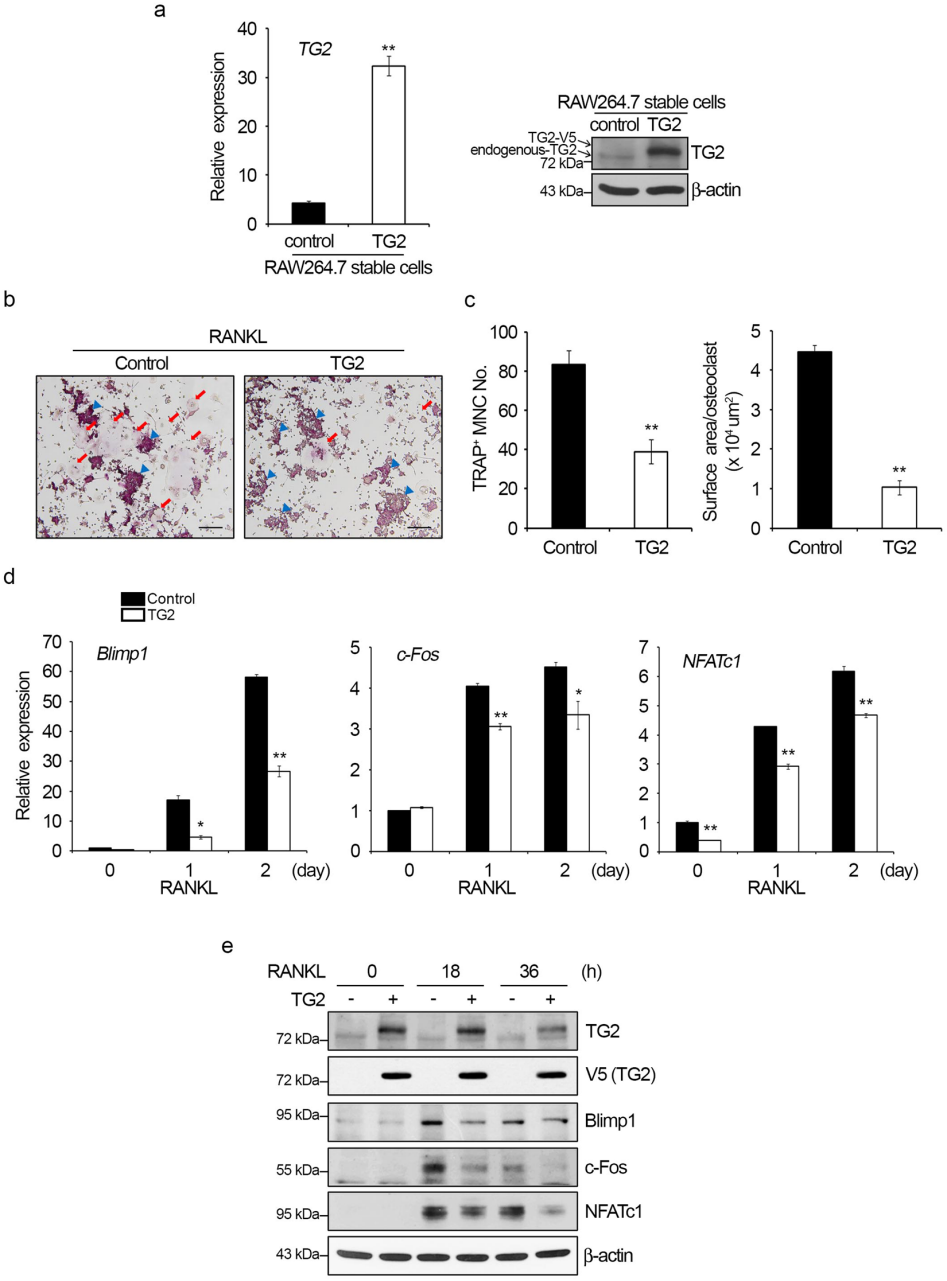
TG2 overexpression inhibits osteoclast formation and Blimp1 expression in RAW264.7 cells. TG2-overexpressing RAW264.7 cells were generated by lentiviral transduction. (**a**) *TG2* mRNA levels in the control and TG2-overexpressing cells were measured by real-time PCR (left panel). **P < 0.005 versus control stable RAW264.7 cells. Whole cell lysates were subjected to Western blot analysis with anti-TG2 antibody (right panel). (**b**) The control and TG2-overexpressing RAW264.7 cells were cultured with RANKL for four days, and TRAP staining was performed. Red arrows indicate MNCs. Blue arrowheads indicate mononucleated cells. Scale bar, 100 μm. (**c**) TRAP-positive MNCs were counted, and the surface area per osteoclast was measured. **P < 0.005 versus control stable RAW264.7 cells. (**d**) The mRNA levels were measured by real-time PCR with normalization to the level of *HPRT*. *P < 0.05; **P < 0.005 versus control stable RAW264.7 cells. (**e**) Whole cell lysates were subjected to Western blot analysis using the indicated antibodies. Anti-V5 antibody was used to detect ectopic TG2. Full length Western blots are presented in Supplementary Figure 10.

We next investigated the role of TG2 in osteoclastogenesis by evaluating the effects of TG2 overexpression in RAW264.7. Using a lentiviral system, a TG2-overexpressing RAW264.7 subline was established. The mRNA and protein levels of TG2 were much higher in these TG2-transduced cells (Fig. 5a). When these cells were induced to differentiate with RANKL, osteoclast formation was less active than in control cells (Fig. 5b). Both the total number of TRAP-positive MNCs and the surface area per osteoclast were significantly lowered by TG2 overexpression (Fig. 5c). Consistently, TG2 overexpression reduced the expression of *Blimp1*, *c-Fos* and *NFATc1* at both mRNA (Fig. 5d) and protein levels (Fig. 5e). These results support that TG2 plays a negative role in osteoclast differentiation via the Blimp1-NFATc1 axis.

### TG2 knockout (KO) mice have decreased bone mass

**Figure 6:**
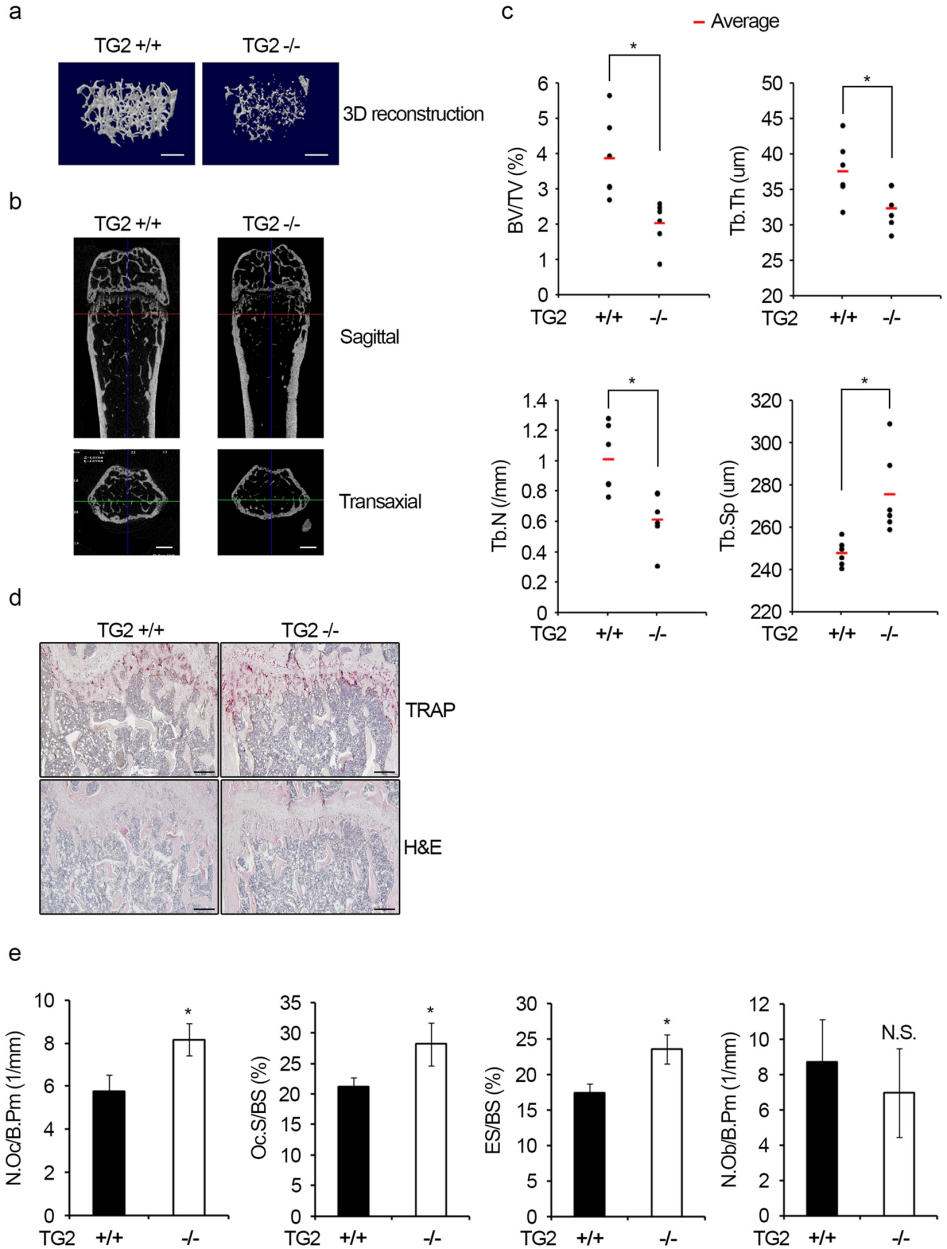
TG2 deficiency decreases trabecular bone mass. (**a**,**b**) Three-dimensional micro-CT images of the distal femurs of 13-week-old WT and *TG2* KO mice (n = 6 for each group) were reconstructed with Skyscan CTvol software. The sagittal and transaxial images generated with DataViewer software are shown. Scale bar, 500 μm. (**c**) The trabecular bone volume per tissue volume (BV/TV), trabecular thickness (Tb.Th), trabecular number (Tb.N), and trabecular separation (Tb.Sp) in distal femurs were quantified by micro-CT analysis with Skyscan CTAn software. (**d**) Decalcified sections of distal femurs of WT and *TG2* KO mice were stained for TRAP activity or with hematoxylin/eosin (H&E). Scale bar, 100 μm. (**e**) The number of osteoclasts per bone perimeter (N.Oc/B.Pm), osteoclast surface per bone surface (Oc.S/BS), eroded surface per bone surface (ES/BS), and the number of osteoblasts per bone perimeter (N.Ob/B.Pm) were quantified. *P < 0.05 versus WT mice. NS, not significant.

To obtain *in vivo* evidence to support a role of TG2 in the regulation of osteoclastogenesis and bone metabolism, we analyzed the radiological and histomorphometric parameters of *TG2* KO mice bones. Three-dimensional micro-CT analyses of femurs from 13-week-old female mice revealed that the trabecular bone volume was significantly lower in *TG2* KO mice compared with wild-type (WT) mice (Fig. 6a). Similarly, sagittal and transaxial images of femurs showed reduction in trabecular bone mass in *TG2* KO mice (Fig. 6b). The trabecular bone volume per tissue volume (BV/TV) was 52% lower in *TG2* KO mice compared with WT mice (Fig. 6c). The trabecular thickness (Tb.Th) and trabecular number (Tb.N) were also decreased in *TG2* KO mice (Fig. 6c). Consistently, *TG2* KO mice had a significantly higher level of trabecular separation (Tb.sp) compared with WT mice (Fig. 6c). To determine whether the decreased bone mass in *TG2* KO mice was related to increased osteoclastogenesis, we analyzed bone tissue sections stained for TRAP activity or with hematoxylin and eosin (H&E) after demineralization (Fig. 6d). The number of osteoclasts per bone perimeter (N.Oc/B.Pm) and the level of osteoclast surface per bone surface (Oc.S/BS) were higher in *TG2* KO versus WT mice (Fig. 6e). The eroded surface per bone surface (ES/BS) was also higher in *TG2* KO mice (Fig. 6e). These results suggest that TG2 deficiency increased the generation of osteoclasts competent to perform the resorption function. As previous reports have implicated a positive role of some TG isoforms in osteoblast differentiation^28, 30^, we also analyzed whether the low bone mass phenotype of *TG2* KO mice was associated with reduced *in vivo* osteoblastogenesis. The number of osteoblasts per bone perimeter (N.Ob/B.Pm) was not significantly different between WT and *TG2* KO mice (Fig. 6e). Taken together, these observations indicate that TG2 affects the generation of osteoclasts, but not osteoblasts, in mice.

### Osteoclastogenic potential of TG2 KO BMMs is higher than that of WT BMMs

**Figure 7:**
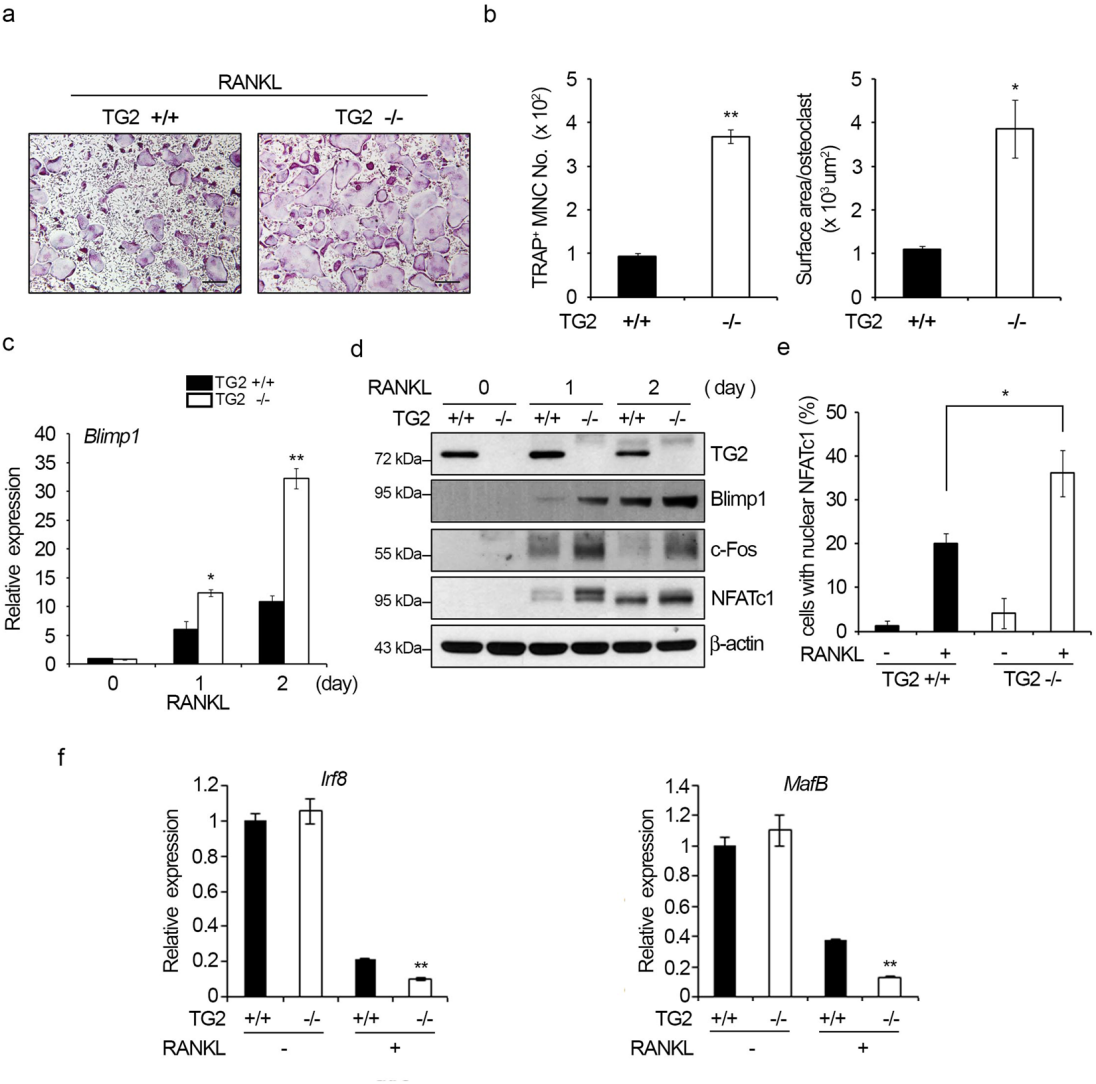
TG2-deficient BMMs show enhanced osteoclast differentiation *in vitro*. (**a**,**b**) BMMs obtained from WT and *TG2* KO mice were cultured with M-CSF (30 ng/ml) and RANKL (100 ng/ml) for three days. Cells were then stained for TRAP. Scale bar, 100 μm. TRAP-positive MNCs were counted, and the surface area per osteoclast was measured. *P < 0.05; **P < 0.005 versus WT cells. (**c**,**d**) BMMs were cultured with M-CSF and RANKL for the indicated number of days. The mRNA levels of *Blimp1* were determined by real-time PCR. *P < 0.05; **P < 0.005 versus WT cells. Cell lysates were analyzed by Western blotting with the indicated antibodies. (**e**) BMMs from WT and *TG2* KO mice were treated with M-CSF and RANKL for two days. Cells were stained for NFATc1 and laminB. Anti-laminB staining marked the nuclear envelope. Cells with nuclear NFATc1 staining were counted. Scale bar, 20 μm. *P < 0.05 versus WT cells. (**f**) BMMs were cultured with M-CSF and RANKL for one day. The mRNA levels of *Irf8* and *MafB* were determined by real-time PCR. *P < 0.05; **P < 0.005 versus WT cells. Full length Western blots are presented in Supplementary Figure 10.

We next investigated whether the reduced bone mass and increased osteoclastogenesis in *TG2* KO mice were due to a cell autonomous difference in osteoclast precursors by comparing the osteoclastogenic potential of BMMs from *TG2* KO and WT mice. The generation of osteoclasts from *TG2* KO BMMs was about 4-fold higher than that from WT BMMs in the *in vitro* differentiation cultures (Fig. 7a). Both the number of TRAP-positive MNCs and the surface area per osteoclast were higher in *TG2* KO cell cultures (Fig. 7b). The number of actin ring-positive cells and the sealing zone area per osteoclast were also higher in *TG2* KO cells (Supplementary Figure 5). The cell proliferation rate was not different between WT and KO BMMs (Supplementary Figure 6). Consistent with the result of the siRNA experiments shown in Fig. 3, an enhancement of Blimp1, c-Fos, and NFATc1 was observed in *TG2* KO cells (Fig. 7c and d). We further examined the activation of NFATc1 by evaluating the intranuclear localization of NFATc1 in osteoclasts. Treatment with RANKL increased nuclear detection of NFATc1, and the percentage of cells with nuclear NFATc1 was higher in *TG2* KO osteoclasts (Fig. 7e and Supplementary Figure 7). Conversely, the expression of IRF8 and MafB was lower in *TG2* KO cells (Fig. 7f). Collectively, these observations indicate that *TG2* KO potentiates osteoclastogenesis by enhancing Blimp1-dependent NFATc1 activation in response to RANKL stimulation.

### TG2 deficiency increases the activation of NF-κB signaling

**Figure 8:**
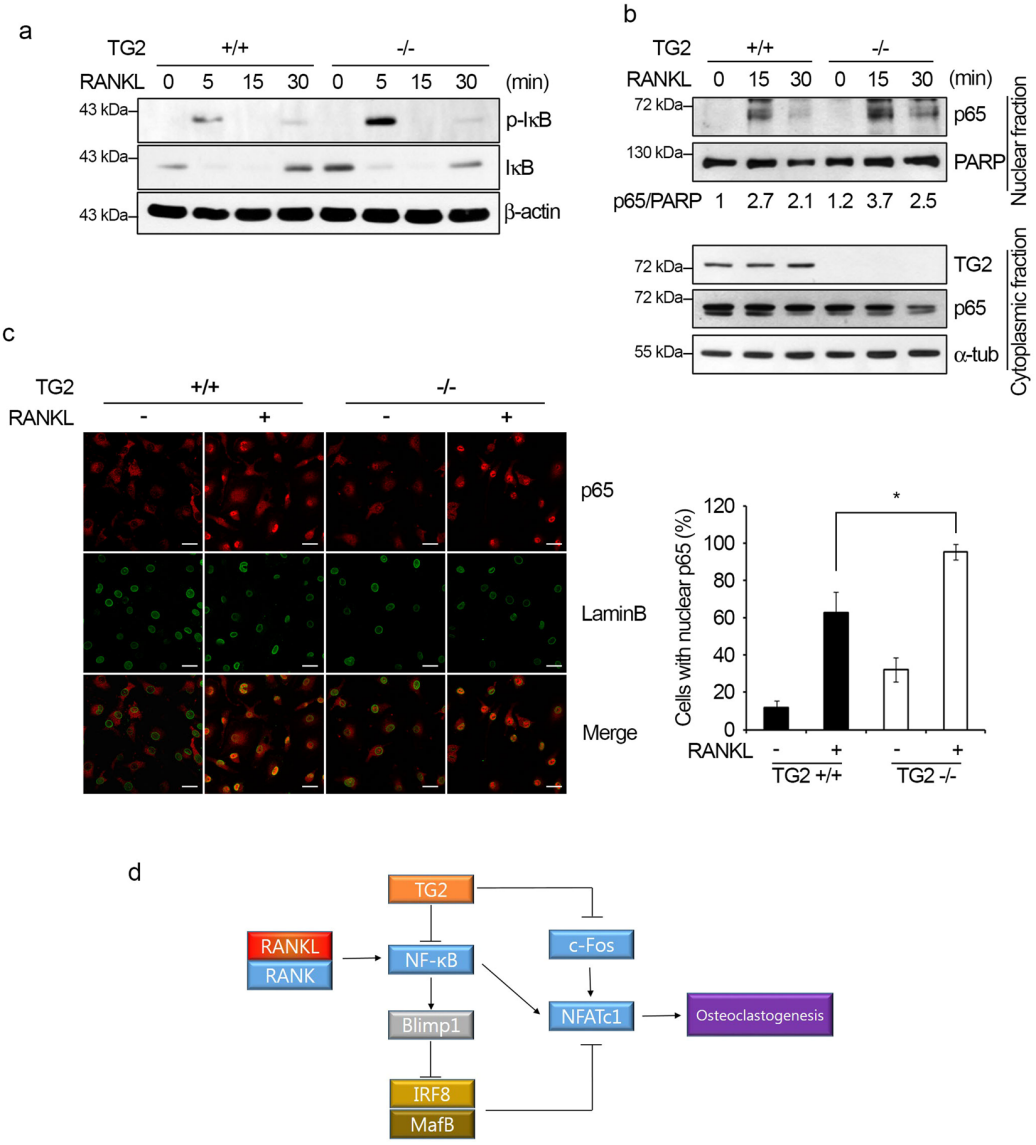
TG2 deficiency augments the activation of the NF-κB signaling pathway by RANKL. (**a**) WT and *TG2* KO BMMs were serum-starved for 5 h and stimulated with RANKL (500 ng/ml) for the indicated times. Whole cell lysates were analyzed by Western blot using anti-phospho-IκB and -IκB antibodies. (**b**) Nuclear and cytosolic fractions prepared from WT and *TG2* KO BMMs were stimulated with RANKL and subjected to Western blot analysis. PARP (full length) and α-tubulin were used as loading controls for nuclear and cytoplasmic extracts, respectively. The ratios of p65 to PARP were determined by densitometry. (**c**) WT and *TG2* KO BMMs were serum-starved for 5 h and stimulated with or without RANKL (500 ng/ml) for 15 min. Cells were stained for p65 and laminB. Anti-laminB staining marked the nuclear envelope. Cells with nuclear p65 staining were counted. Scale bar, 20 μm. *P < 0.05 versus WT cells. (**d**) Schematic illustration of a proposed mechanism through which TG2 regulates osteoclastogenesis. A reduction in TG2 enhances the activation of NF-κB p65, which increases the expression of Blimp1. Blimp1 together with c-Fos augments the expression and nuclear translocation of NFATc1 to enhance osteoclastogenesis induced by RANKL. Full length Western blots are presented in Supplementary Figure 10.

To further validate the target signaling pathways affected by TG2 deficiency, we evaluated the activation of the NF-κB pathway in WT and *TG2* KO BMMs. RANKL-induced IκB phosphorylation was higher in *TG2* KO BMMs than in WT BMMs 5 min after stimulation (Fig. 8a). The level of total IκB was lower in *TG2* KO BMMs 30 min after RANKL stimulation (Fig. 8a). In addition, nuclear p65 level increased upon RANKL stimulation at 15 and 30 min, and this increase was greater in *TG2* KO BMMs than in WT BMMs (Fig. 8b). Nuclear translocation of p65 was also assessed by confocal microscopy. As shown in Fig. 8c, *TG2* KO BMMs had a higher percentage of positive cell for nuclear p65 compared to WT BMMs. This observation indicates that p65 translocation to the nucleus in response to RANKL was enhanced by TG2 deficiency. The MAPK activation by RANKL was not affected by the absence of TG2 (Supplementary Figure 8). These results from *TG2* KO BMMs, together with the data from *TG2* knockdown cells, support that the NF-κB signaling pathway is crucial for TG2-mediated regulation of osteoclast differentiation.

## Discussion

Previous studies have suggested that TG activity is involved in the regulation of cell adhesion, matrix deposition, and differentiation of osteoblasts^28–30^. However, the relationship between TGs and osteoclast regulation has not been investigated. In this study, we explored the functional role of the TG2 isoform in osteoclast differentiation. Our *in vitro* and *in vivo* experiments revealed that TG2 negatively regulates osteoclastogenesis via a NF-κB- and Blimp1-dependent pathway.

Among the eight TG family members, TG2 was selectively expressed in osteoclasts (Fig. 1a). The reduction in TG2 level by TG2-targeted siRNA enhanced RANKL-dependent osteoclast differentiation *in vitro* and increased induction of c-Fos, NFATc1, and Blimp1 by RANKL (Figs 1 and 3). This negative association between TG2 level and osteoclastogenesis was confirmed using *TG2* KO mice that displayed increased numbers of osteoclasts on the bone surface and less trabecular bone mass than WT mice (Fig. 6). Conversely, TG2 overexpression in RAW264.7 cells suppressed osteoclastogenesis, further supporting the inhibitory role of TG2 in osteoclast differentiation (Fig. 5). The inhibitory function of TG2 despite apparent up-regulation by RANKL at early stages (Fig. 3a and b) suggests that TG2 forms a negative feedback loop to fine-tune osteoclastogenesis, preventing excessive osteoclast differentiation and activation. This type of negative feedback regulation in osteoclastogenesis has been reported from our and other groups^35, 36^.

In this study, we found that Blimp1 is an important mediator of TG2 function in the regulation of osteoclastogenesis. Blimp1 was first identified as a transcriptional repressor of the interferon beta promoter and has been characterized to play crucial roles in immune cell development, including the terminal differentiation of B-cells to plasma cells^13^. More recently, Blimp1 was also shown to be involved in the regulation of osteoclastogenesis through down-regulation of anti-osteoclastogenic transcription factors like IRF8 and MafB, which inhibit RANKL induction of NFATc1^14^. In addition, inhibition of Bcl6, which suppresses the expression of NFATc1, DC-STAMP, and cathepsin K, by Blimp1 was suggested to contribute to efficient osteoclast differentiation^37^. The first clue to the relationship between Blimp1 and TG2 was obtained in a study of lymphocytes^33^. In that study, spleen cells from *TG2* KO mice showed a higher percentage of plasma cells and elevated level of *Blimp1* mRNA in response to immunization^33^, implying a negative regulation of Blimp1 expression by TG2. However, the potential mechanisms by which TG2 regulates Blimp1 expression have not been elucidated to date.

The regulation of Blimp1 expression by TG2 in RANKL-treated BMMs appeared to occur at the level of transcription (Figs 3a, 5d and 7c). This transcriptional regulation of Blimp1 is consistent with the results of a study using spleen B cells from *TG2* KO mice^33^. TG2-mediated cross-linking and the subsequent inactivation of transcription factors, such as Sp1, have been suggested as the mechanism by which TG2 down-regulates gene expression^38^. Another mechanism of TG2-dependent transcriptional inhibition involves AP1; in this mode, TG2 interacts with c-Jun and consequently reduces dimmer formation between c-Jun and c-Fos^39^. We cannot exclude the possibility of an analogous means of direct inhibition by TG2 of the binding and/or activation of transcription factors that target the Blimp1 promoter. However, our Western blot results did not show the presence of TG2 protein in nuclear fractions (Fig. 3c), suggesting that other mechanisms are more likely to be responsible for the increase in Blimp1 caused by TG2 deficiency. Indeed, in our study, RANKL-induced phosphorylation of IκB was potentiated by TG2 knockdown (Fig. 3d), and an NF-κB inhibitor abrogated Blimp1 up-regulation by TG2 knockdown (Fig. 3h). Therefore, it is possible that TG2 inhibits a kinase that phosphorylates IκB, perhaps IKK, or stimulates a phosphatase that targets phosphorylated IκB in the cytosol. In this regard, the function of TG2 as a GTPase signaling molecule^40^ might be of particular interest. Further investigations will be required to reveal the precise mechanism accounting for this novel TG2 action on the NF-κB signaling pathway.

c-Fos also appears to be involved in the regulation of osteoclastogenesis by TG2. In TG2- deficient BMMs, the extent of c-Fos induction by RANKL was stronger relative to that of control cells (Figs 1, 3, 4 and 7). Conversely, TG2 overexpression led to weaker c-Fos induction by RANKL (Fig. 5). This effect of TG2 on c-Fos expression was not affected by NF-κB inhibition (Fig. 3h). In addition, the effect of Blimp1 knockdown on c-Fos expression was minimal (Fig. 4d). Therefore, the regulation of c-Fos by TG2 is likely to be independent of the NF-κB-Blimp1 axis. As c-Fos is a component of the AP1 transcription factor and its expression is positively auto-regulated, the suppressive effect of TG2 on c-Fos induction might be attributed to the aforementioned mechanism of interaction of TG2 with c-Jun and the resulting reduction in AP1 dimer formation. Although the identification of the direct targets of TG2 required for regulation of c-Fos and NF-κB was beyond the scope of this study, we clearly demonstrated that two separate pathways involving NF-κB and Blimp1 on one axis and c-Fos on the other axis mediated the effect of TG2 on osteoclastogenesis (Fig. 8d).

In our study, *TG2* KO mice showed increased numbers of osteoclasts on the bone surfaces compared with WT mice. However, the number of osteoblasts was not significantly different between *TG2* KO and WT mice (Fig. 6e). Therefore, the lower bone mass of *TG2* KO mice is likely to be due to the higher bone-resorbing activity of osteoclasts rather than to lower bone-forming activity by osteoblasts. This observation might appear to be inconsistent with previous reports where TG enzymatic activities were implicated in matrix mineralization and osteoblastic differentiation^29, 30^. However, clear evidence supporting the contribution of TGs, specifically the TG2 isoform, to bone formation or osteoblast differentiation has not been provided to date. Indeed, *TG2* KO mice did not show any evident defect in bone development upon macro- and microscopic examination^41^. The normal skeletal development of *TG2* KO mice has been suggested to be due to compensatory overexpression of FXIIIA and TGF-β^42^. While it remains to be determined whether FXIIIA compensates for the lack of TG2 activity during osteoblastogenesis in *TG2* KO mice, TG2 deficiency was not compensated for osteoclastogenesis in our study (Figs 6 and 7). The lack of expression of other TG isoforms, with only fairly weak expression of FXIIIA, during osteoclastogenesis (Fig. 1a) might explain the overt effect of TG2 deficiency on osteoclasts but not on osteoblasts.

Our present study revealed for the first time a regulatory role of TG2 in osteoclast differentiation. We further demonstrated that this TG2 function was mediated by the NF-κB-Blimp1 and c-Fos pathways that converge on NFATc1. Further investigation of the direct target molecules of TG2 and the mode of TG2 action on the targets will be required to comprehensively understand the role of TG2 associated with the regulation of osteoclastogenesis and bone metabolism.

## Materials and Methods

### Reagents

Re combinant RANKL and M-CSF were purchased from Peprotech EC Ltd. (London, UK). Anti-TG2, anti-c-Fos, anti-NFATc1, anti-α-tubulin, and the NF-κB inhibitor BAY 11-7085 were purchased from Santa Cruz Biotechnology (Santa Cruz, CA, USA). Anti-Blimp1 was purchased from Abcam (Cambridge, UK). Polyclonal antibodies against ERK, JNK, p38, IκB, p65, phospho-ERK, phospho-JNK, phospho-p38, phospho-IκB, phospho-p65, and PARP were purchased from Cell Signaling Technology (Cambridge, MA, USA). A monoclonal antibody against β-actin and horseradish peroxidase (HRP)-conjugated secondary antibodies were from Sigma Aldrich (St Louis, MO, USA). NFATc1 and NF-κB p65 Transcription Factor Assay Kits were purchased from Active Motif (Carlsbad, CA, USA). Cell Counting Kit-8 (CCK) was obtained from Dojindo (Kumamoto, Japan). The NE-PER Nuclear and Cytoplasmic Extraction Reagents Kit was obtained from Pierce Biotechnology (Rockford, IL, USA). The TRAP staining kit and other reagents were purchased from Sigma Aldrich. Control, TG2 (#1) and Blimp1 siRNAs were purchased from Invitrogen (Carlsbad, CA, USA). Control, TG2 (#2), c-Fos and NFATc1 siRNAs were purchased from Bioneer (Daejun, Korea). The sequences of oligonucleotides were 5′-CAGTTCGAGGATGGAATCCTGGATA-3′ for TG2 siRNA (#1), 5′- GAGGCCGAGTTTGAAGAGAAGTGTA for Blimp1 siRNA,. 5′-TCACACAGTGCCTAGACTA(dTdT)-3′ for TG2 siRNA (#2), 5′-CAGATCTGTCCGTCTCTAG(dTdT)-3′ for c-Fos siRNA, and 5′-CTCTGTGGCCCTCAAAGTA(dTdT)-3′ for NFATc1 siRNA.

### Animals

*TG2* KO mice have been previously described^41^ and were bred at the SPF animal facility of Seoul National University School of Dentistry. The ICR mice used for the preparation of osteoclast precursor cells were purchased from OrientBio (Seongnam, Korea). All animal experiments were conducted according to the Guidelines for Animal Experimentation and performed with the approval of the Institutional Animal Care and Use Committee at Seoul National University.

### Bone marrow-derived macrophages (BMMs)

Mouse BMMs were prepared as previously described^43^. Bone marrow from the tibiae and femurs of 5-week-old mice were flushed with α-MEM (Welgene, Daegu, Korea). After removing erythrocytes with hypotonic buffer, cells were cultured in α-MEM containing 10% FBS for 24 h, and adherent cells were discarded. Non-adherent bone marrow cells were plated onto 100-mm non-coated Petri dishes at a density of 5 × 10^6^ cells per dish and cultured in the presence of M-CSF (30 ng/ml) for three days. Adherent cells at this stage were considered to be bone marrow-derived macrophages (BMMs) and were used as osteoclast precursors.

### Cell culture

BMMs were cultured in α-MEM supplemented with 10% (vol/vol) heat-inactivated FBS, 100 units/ml penicillin, and 100 μg/ml streptomycin. BMMs were plated in 60-mm dishes at 5 × 10^5^ cells per dish or in 48-well culture plates at 4 × 10^4^ cells per well. RAW264.7 and 293FT cells were cultured in DMEM supplemented with 10% heat-inactivated FBS, 100 units/ml penicillin, and 100 μg/ml streptomycin. RAW264.7 cells were plated in 48-well culture plates at 1 × 10^4^ cells per well, and 293FT cells were plated in 60-mm culture dishes at 4 × 10^5^ cells per dish.

### Chromatin immunoprecipitation assay

RAW264.7 cells transfected with control siRNA or TG2 siRNA were cultured in the presence of RANKL (100 ng/ml) for 24 h. After serum-starvation for 5 h, cells were stimulated with RANKL (500 ng/ml) for 15 min. Cells were collected for chromatin immunoprecipitation assay following the manufacturer’s protocol (Millipore, Billerica, MA, USA). Briefly, cells were crosslinked with 37% formaldehyde for 10 min and unreacted formaldehyde was quenched by glycine. SDS lysis buffer containing protease inhibitor cocktail was used to lyse cells, followed by sonication to shear DNA. Chromatin lysates were precleared with Protein G Agarose for 1 h and incubated with p65 antibody (1:200) or control rabbit IgG overnight at 4 °C with rotation. Next day, the antibody/antigen/DNA complexes were collected by Protein G agarose incubation for 1 h. The complexes were eluted with 1% SDS and 0.1 M NaHCO_3_ and reverse crosslinked with NaCl (overnight incubation at 65 °C). DNA was purified using spin columns and PCR was performed (32 cycles) using primers covering putative binding sites of p65 on the Blimp1 promoter. All primer sets are listed in Supplementary Table 1.

### Preparation of cytoplasmic and nuclear proteins

Cytoplasmic and nuclear proteins were prepared from BMMs using the NE-PER Nuclear and Cytoplasmic Extraction Reagents Kit according to the manufacturer’s instructions. In brief, cultured cells were washed with PBS and treated with the cell lysis buffer provided in the kit. After centrifugation, the supernatant was transferred to a new tube as cytoplasmic extract. The pellet was washed twice and lysed with the nuclear lysis reagent provided in the kit. After centrifugation, the supernatant was used as the nuclear protein extract. Protein concentrations were determined using a detergent-compatible colorimetric assay kit (Bio-Rad Laboratories, Hercules, CA, USA).

### RT-PCR and real-time PCR

After isolation of total RNA using TRIZOL (Invitrogen), reverse transcription was performed using 2 μg of total RNA and Superscript II reverse transcriptase (Invitrogen) according to the manufacturer’s protocol. PCR was performed using 26 cycles of denaturation at 95 °C for 15 sec and annealing/extension at 60 °C for 60 sec. PCR products were separated in 1.5% agarose gels and stained with ethidium bromide. The *HPRT* housekeeping gene was used as an input control. Real-time PCR was performed using the ABI 7500 real-time system with the KAPA SYBR FAST qPCR kit (Kapa Biosystems, Wilmington, MA, USA). The detector was programmed with the following PCR conditions: 40 cycles of 3 sec denaturation at 95 °C and 33 sec amplification at 60 °C. All reactions were run in triplicate. The mRNA levels of target genes were normalized to the mRNA level of the *HPRT* gene, and values indicating the fold-changes from the controls are shown. Relative differences in mRNA levels were evaluated using the comparative cycle threshold method. All primer sets for RT-PCR and real-time PCR are listed in Supplementary Table 2.

### Gene cloning and lentiviral transduction

To generate TG2-overexpressing stable RAW264.7 cells, the 2061 bp cDNA (GenBank accession number NM_009373.3) fragment of *TG2* was obtained by RT-PCR of mRNA from BMMs. The PCR reaction steps included an initial denaturation at 95 °C for 5 min, followed by reaction cycles of denaturation at 95 °C for 30 sec, annealing at 56 °C for 30 sec, and extension at 72 °C for 4 min. The following primers were used: *TG2*, 5′-GGACTAGTGCCACCATGGCAGAGGAGCTGCTC-3′ (sense) and 5′-TCCCCGCGGGGCCGGGCCGATGATAAC-3′ (antisense). The pLenti6 lentiviral vector and the *TG2* PCR product were cleaved with SpeI and SacII restriction enzymes and subjected to 1% agarose gel electrophoresis. Each DNA fragment was purified from the gel, and the *TG2* fragment was ligated to the pLenti6 vector DNA. To generate lentiviral particl es, 293FT cells were transfected with pLenti6-LacZ (control) or pLenti6-TG2 using PolyFect Transfection Reagent (Qiagen, Leipzig, Germany). Culture supernatant containing viral particles was harvested after 48 h and passed through a 0.45 μm syringe filter. RAW264.7 cells were infected with viral supernatant mixed with polybrene (6 μg/ml) for 18 h. After an overnight incubation, infected RAW264.7 cells were selected with blasticidin (1:2000) for 15 days. Overexpression of TG2 was determined by measuring mRNA and protein levels.

### Western blotting

BMMs and RAW264.7 cells were lysed with a lysis buffer containing 120 mM Tris–HCl (pH 7.5), 150 mM NaCl, 1 mM EDTA, 1 mM EGTA, 0.5% NP40, 2.5 mM sodium pyrophosphate, 1 mM β-glycerophosphate, 1 mM Na_3_VO_4_, 1 mM NaF, and protease inhibitors (Roche, Mannheim, Germany). The protein concentrations of cell lysates were determined using the DC protein assay kit (Bio-Rad), and equal amounts of protein were loaded onto 10% or 12% SDS-polyacrylamide gels. After transfer onto nitrocellulose membranes (Amersham Pharmacia, Uppsala, Sweden) and blocking with 5% nonfat skim milk, primary antibodies were added, and membranes were incubated overnight at 4 °C. After washing, the membranes were incubated with HRP-conjugated secondary antibodies in 2% skim milk for 1 h. Immunoreactivity was detected with ECL reagents. Comparable loading was verified by reprobing the same membranes with anti-β-actin antibody.

### TRAP staining and osteoclast measurement

To generate osteoclasts, BMMs and RAW 264.7 cells were plated in 48-well tissue culture plates at densities of 4 × 10^4^ cells/well and 1 × 10^4^ cells/well, respectively. Cells were cultured in the presence or absence of RANKL (100 ng/ml) for 3 or 4 days. BMM cultures were supplemented with M-CSF (30 ng/ml). The culture medium was changed at day 2. Under these conditions, TRAP-positive mononuclear cells were detected at day 2, and TRAP-positive MNCs ($\ge$ 3 nuclei) were observed at days 3 and 4. The cells were fixed with 3.7% formaldehyde for 30 min at room temperature. After permeabilization with 0.1% Triton X-100 for 5 min, TRAP staining was performed in the dark for 30 min using a TRAP-staining kit following the manufacturer’s instructions. After staining, cells were washed with distilled water and observed under a light microscope. The total number of TRAP-positive MNCs and the surface area per osteoclast were measured using Osteomeasure software (OsteoMetrics, Inc., Decatur, GA, USA).

### Resorption assay

Bone resorption assays were performed as previously described^44^. Briefly, BMMs seeded on dentine discs (Osteosite Dentine Discs, Immunodiagnostic Systems Inc., Boldon, UK) were stimulated with RANKL for seven days. After removing cells by treating the dentine discs with 5% sodium hypochlorite for 10 min and wiping with cotton swab, the discs were photographed. The areas and depths of the resorption pits were measured with a Carl Zeiss LSM 5-PASCAL laser-scanning microscope (Carl Zeiss Microimaging GmbH, Goettingen, Germany).

### Confocal microscopy

BMMs were seeded on glass cover slips in 24-well plates at the density of 7 × 10^4^ cells per well. Cultured cells were fixed with 3.7% formaldehyde and permeabilized with 0.1% Triton X-100. After blocking in PBS containing 1% BSA, the cover slips were incubated with a suitable primary antibody (1:100) overnight at 4 °C. Subsequently, cells were washed and incubated with FITC- or Cy3-conjugated secondary antibodies (1:250) for 1 h at room temperature. For sealing zone formation assays, BMMs were cultured in the presence of M-CSF (30 ng/mL) and RANKL (100 ng/ml) in α-MEM supplemented with 10% FBS. After three days in culture, mature osteoclasts were fixed and stained using rhodamine-phalloidin for actin and 4′,6-diamidino-2-phenylindole (DAPI) for nuclei. The mean intensity of sealing zone per osteoclast was measured using Zen 2009 software (Carl Zeiss). For the p65 nuclear translocation assay, BMMs were serum-starved for 5 h and stimulated with RANKL (500 ng/ml) for the indicated lengths of time. Stimulated cells were fixed and subjected to immunocytochemistry using anti-laminB and anti-p65 antibodies. For the NFATc1 nuclear translocation assay, BMMs were cultured in the presence of M-CSF (30 ng/mL) and RANKL (100 ng/ml). After two days in culture, cells were fixed and stained using an antibody against NFATc1, laminB, or TG2. The cover slips were mounted, and cell images were obtained using a Carl Zeiss LSM 700 confocal microscope.

### Three-dimensional micro-computed tomography analysis

Femurs of 13-week-old female *TG2* KO mice and WT littermate mice (6 per genotype) were fixed in 4% (vol/vol) paraformaldehyde overnight and analyzed by micro-computed tomography using the SkyScan 1072 system (SkyScan, Kontich, Belgium). Trabecular bone parameters were measured in 1-mm thick trabecular regions, starting 1 mm below the proximal edge of the growth plate in the distal end of the femur. A total of 350–400 tomographic slices were acquired and three-dimensional analyses were performed with CT-volume software (ver 2.1.1.0; Skyscan).

### Histomorphometric analysis

Bone histomorphometric analyses were performed using paraffin-embedded sections. In brief, femurs were fixed in 4% paraformaldehyde, decalcified in 12% EDTA for 4 weeks, and embedded in paraffin. The paraffin blocks were serially sectioned at a thickness of 6 μm and stained with TRAP/hematoxylin or hematoxylin/eosin according to standard procedures. Trabecular bone parameters were analyzed using Osteomeasure software.

### Statistical analysis

All experiments, except the *in vivo* KO mouse analysis, were performed three or more times. Data are presented as mean ± SD. Statistical significance was determined using Student’s t test for *in vitro* and *in vivo* studies. *P* values less than 0.05 were considered to be statistically significant.

## Electronic supplementary material

Supplementary Information

## References

[1] Martin TJ, Sims NA (2005). Osteoclast-derived activity in the coupling of bone formation to resorption. Trends Mol Med.

[2] Walsh MC (2006). Osteoimmunology: interplay between the immune system and bone metabolism. Annu Rev Immunol.

[3] Hayashi S (1998). Commitment and differentiation of stem cells to the osteoclast lineage. Biochem Cell Biol.

[4] Boyle WJ, Simonet WS, Lacey DL (2003). Osteoclast differentiation and activation. Nature.

[5] Teitelbaum SL (2000). Bone resorption by osteoclasts. Science.

[6] Lee ZH, Kim HH (2003). Signal transduction by receptor activator of nuclear factor kappa B in osteoclasts. Biochem Biophys Res Commun.

[7] Kim JH, Kim N (2014). Regulation of NFATc1 in Osteoclast Differentiation. J Bone Metab.

[8] Takayanagi H (2002). Induction and activation of the transcription factor NFATc1 (NFAT2) integrate RANKL signaling in terminal differentiation of osteoclasts. Dev Cell.

[9] Wagner EF, Eferl R (2005). Fos/AP-1 proteins in bone and the immune system. Immunol Rev.

[10] Kukita T (2004). RANKL-induced DC-STAMP is essential for osteoclastogenesis. J Exp Med.

[11] Lee SH (2006). v-ATPase V0 subunit d2-deficient mice exhibit impaired osteoclast fusion and increased bone formation. Nat Med.

[12] Georgess D (2014). Comparative transcriptomics reveals RhoE as a novel regulator of actin dynamics in bone-resorbing osteoclasts. Mol Biol Cell.

[13] Martins G, Calame K (2008). Regulation and functions of Blimp-1 in T and B lymphocytes. Annu Rev Immunol.

[14] Nishikawa K (2010). Blimp1-mediated repression of negative regulators is required for osteoclast differentiation. Proc Natl Acad Sci U S A.

[15] Kim K (2007). MafB negatively regulates RANKL-mediated osteoclast differentiation. Blood.

[16] Zhao B (2009). Interferon regulatory factor-8 regulates bone metabolism by suppressing osteoclastogenesis. Nat Med.

[17] Griffin M, Casadio R, Bergamini CM (2002). Transglutaminases: nature’s biological glues. Biochem J.

[18] Barone MV (2007). Humoral immune response to tissue transglutaminase is related to epithelial cell proliferation in celiac disease. Gastroenterology.

[19] Balajthy Z (2006). Tissue-transglutaminase contributes to neutrophil granulocyte differentiation and functions. Blood.

[20] Fesus L, Szondy Z (2005). Transglutaminase 2 in the balance of cell death and survival. FEBS Lett.

[21] Akimov SS, Belkin AM (2001). Cell surface tissue transglutaminase is involved in adhesion and migration of monocytic cells on fibronectin. Blood.

[22] Inada R (2000). Facilitated wound healing by activation of the Transglutaminase 1 gene. Am J Pathol.

[23] Lorand L, Graham RM (2003). Transglutaminases: crosslinking enzymes with pleiotropic functions. Nat Rev Mol Cell Biol.

[24] Quan G, Choi JY, Lee DS, Lee SC (2005). TGF-beta1 up-regulates transglutaminase two and fibronectin in dermal fibroblasts: a possible mechanism for the stabilization of tissue inflammation. Arch Dermatol Res.

[25] Zemskov EA, Janiak A, Hang J, Waghray A, Belkin AM (2006). The role of tissue transglutaminase in cell-matrix interactions. Front Biosci.

[26] Begg GE (2006). Mechanism of allosteric regulation of transglutaminase 2 by GTP. Proc Natl Acad Sci U S A.

[27] Di Venere A (2000). Opposite effects of Ca(2+) and GTP binding on tissue transglutaminase tertiary structure. J Biol Chem.

[28] Nurminskaya M, Kaartinen MT (2006). Transglutaminases in mineralized tissues. Front Biosci.

[29] Nurminskaya M, Magee C, Faverman L, Linsenmayer TF (2003). Chondrocyte-derived transglutaminase promotes maturation of preosteoblasts in periosteal bone. Dev Biol.

[30] Al-Jallad HF (2006). Transglutaminase activity regulates osteoblast differentiation and matrix mineralization in MC3T3-E1 osteoblast cultures. Matrix Biol.

[31] Asagiri M, Takayanagi H (2007). The molecular understanding of osteoclast differentiation. Bone.

[32] Saltel F, Destaing O, Bard F, Eichert D, Jurdic P (2004). Apatite-mediated actin dynamics in resorbing osteoclasts. Mol Biol Cell.

[33] Kim JH (2012). Transglutaminase 2 modulates antigen-specific antibody response by suppressing Blimp-1 and AID expression of B cells in mice. Immunol Lett.

[34] Morgan MA (2009). Blimp-1/Prdm1 alternative promoter usage during mouse development and plasma cell differentiation. Mol Cell Biol.

[35] Kim H, Lee YD, Kim HJ, Lee ZH, Kim HH (2017). SOD2 and Sirt3 Control Osteoclastogenesis by Regulating Mitochondrial ROS. J Bone Miner Res.

[36] Takayanagi H (2002). RANKL maintains bone homeostasis through c-Fos-dependent induction of interferon-beta. Nature.

[37] Miyauchi Y (2010). The Blimp1-Bcl6 axis is critical to regulate osteoclast differentiation and bone homeostasis. J Exp Med.

[38] Tatsukawa H, Kojima S (2010). Recent advances in understanding the roles of transglutaminase 2 in alcoholic steatohepatitis. Cell Biol Int.

[39] Ahn JS (2008). Tissue transglutaminase-induced down-regulation of matrix metalloproteinase-9. Biochem Biophys Res Commun.

[40] Nakaoka H (1994). Gh: a GTP-binding protein with transglutaminase activity and receptor signaling function. Science.

[41] De Laurenzi V, Melino G (2001). Gene disruption of tissue transglutaminase. Mol Cell Biol.

[42] Tarantino U (2009). FXIIIA and TGF-beta over-expression produces normal musculo-skeletal phenotype in TG2−/− mice. Amino Acids.

[43] Chang EJ (2008). Brain-type creatine kinase has a crucial role in osteoclast-mediated bone resorption. Nat Med.

[44] Kim HJ (2012). Plasma membrane calcium ATPase regulates bone mass by fine-tuning osteoclast differentiation and survival. J Cell Biol.

