# Supplementary material for: Transglutaminase 2 regulates osteoclast differentiation via a Blimp1-dependent pathway
Source: Sci Rep. 2017 Sep 6;7:10626. doi: 10.1038/s41598-017-11246-5 (PMC5587636; doi:10.1038/s41598-017-11246-5)
Supplement: Supplementary file 1 — Supplementary Information [file 41598_2017_11246_MOESM1_ESM.pdf]

Transglutaminase 2 regulates osteoclast differentiation via a Blimp1-dependent pathway

Woo-Shin Kim[1*], Haemin Kim[1*], Eui Man Jeong[2], Hyung Joon Kim[3], Zang Hee Lee[1], In-Gyu Kim[2], and Hong-Hee Kim[1]

*Woo-Shin Kim and Haemin Kim contributed equally to this work.

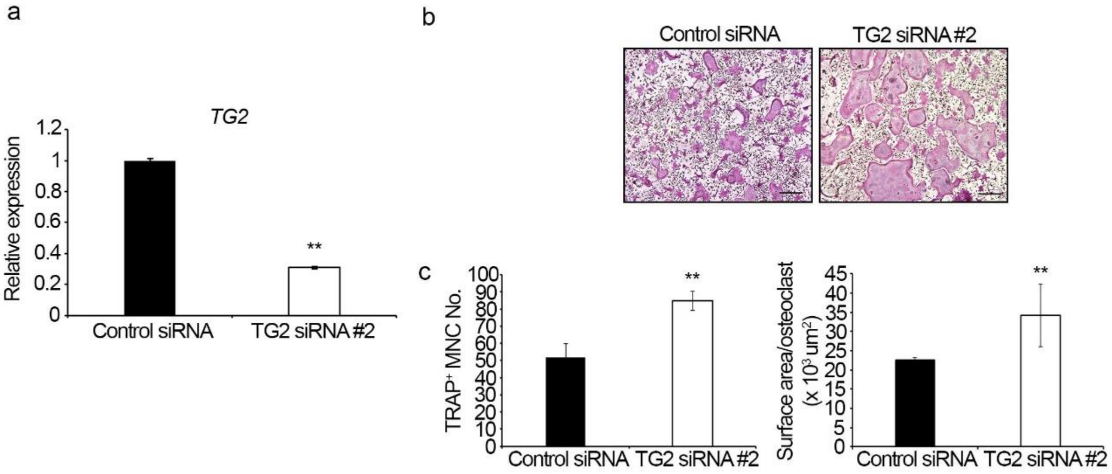

Supplementary Figure 1

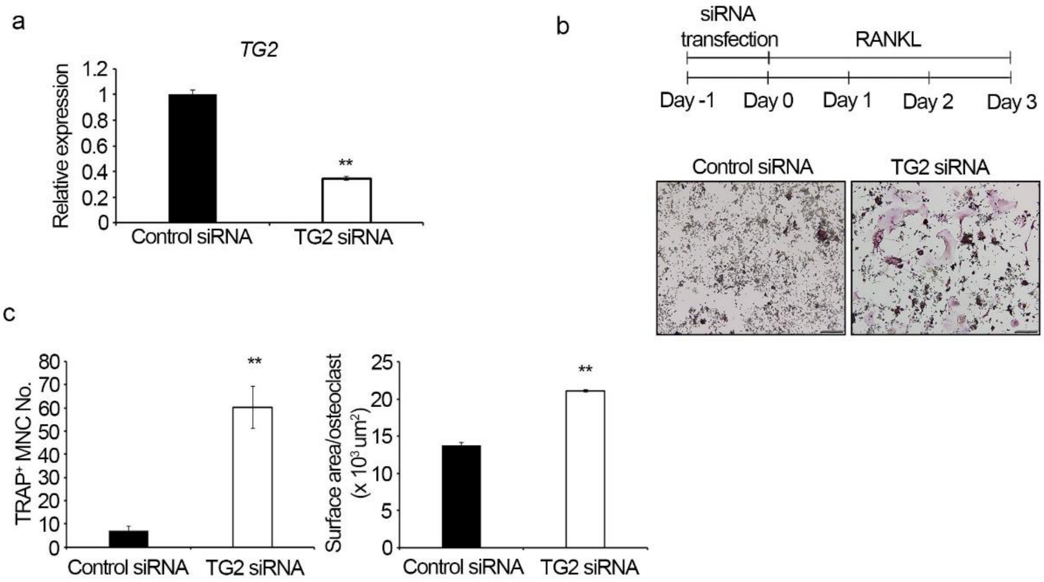

Supplementary Figure 2

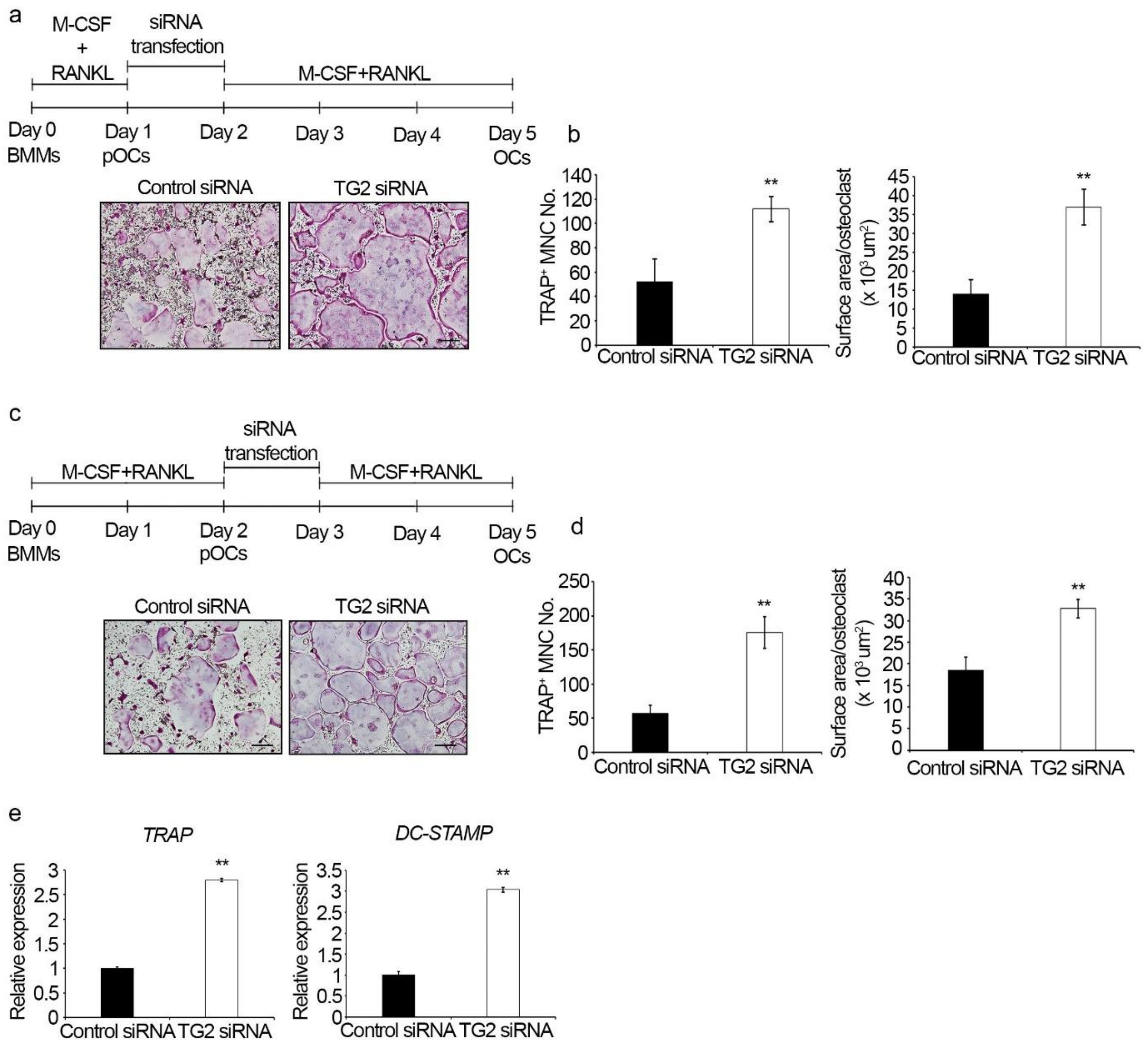

Supplementary Figure 3

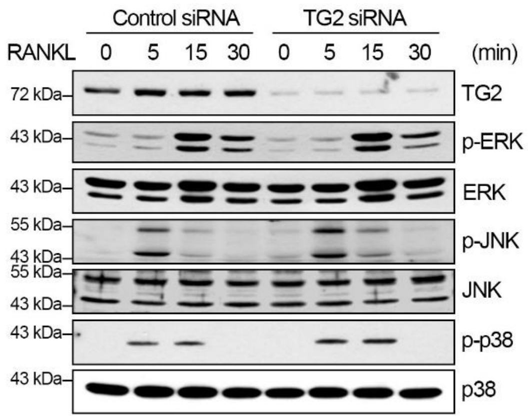

Supplementary Figure 4

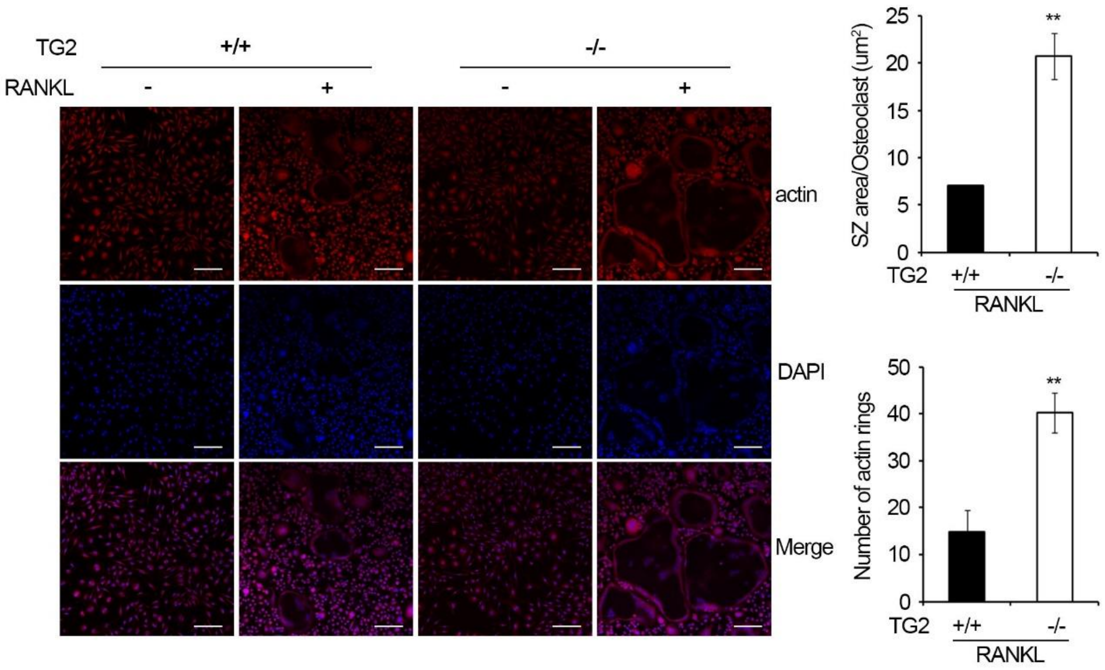

Supplementary Figure 5

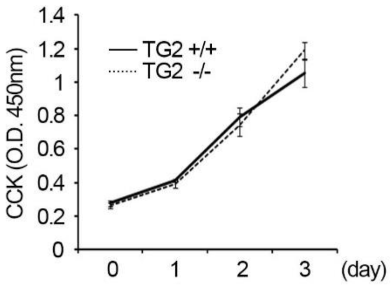

Supplementary Figure 6

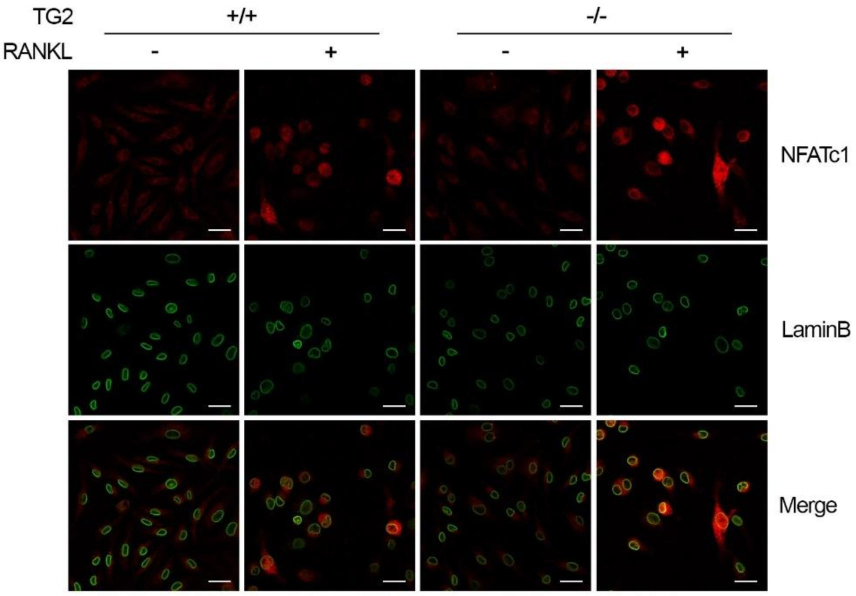

Supplementary Figure 7

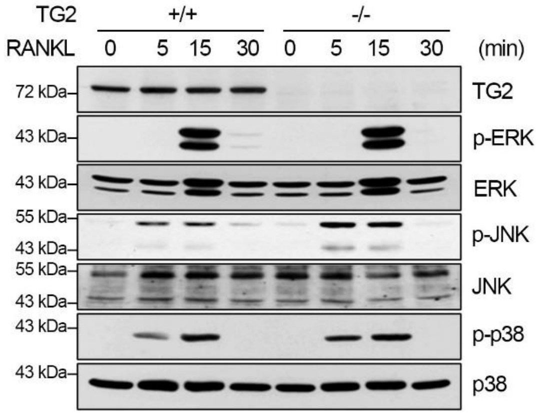

Supplementary Figure 8

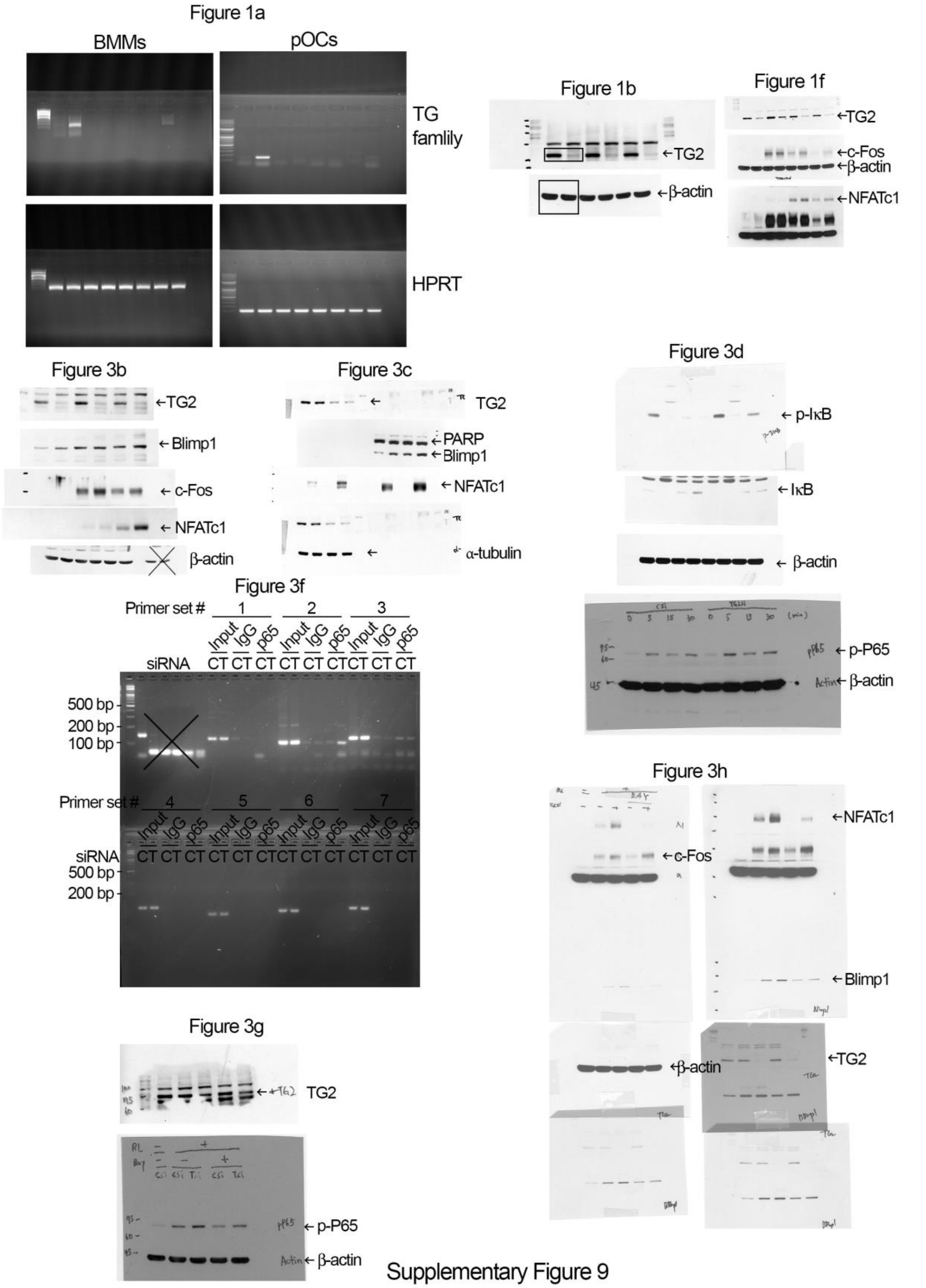

Supplementary Figure 9

Figure 4d

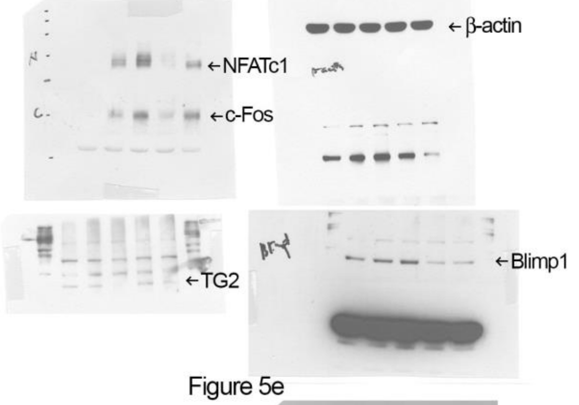

Figure 5a

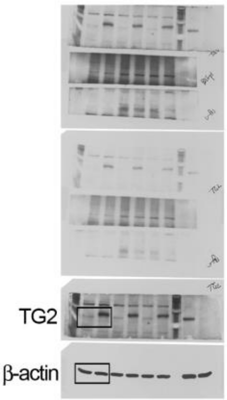

Figure 5e

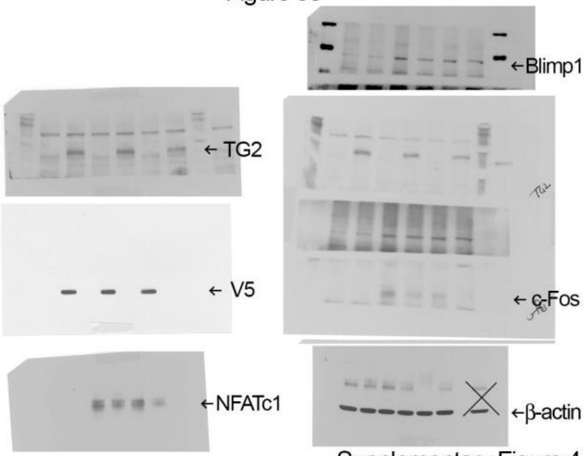

Figure 7d

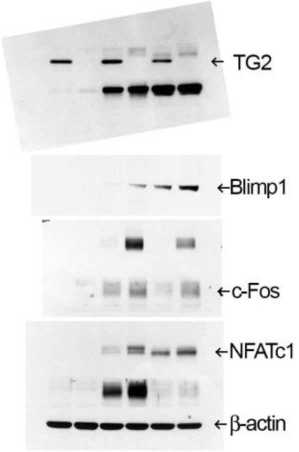

Supplementary Figure 4

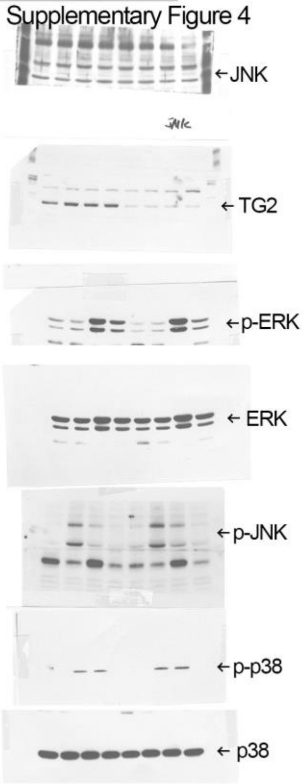

Figure 8a

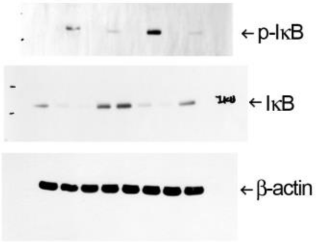

Figure 8b

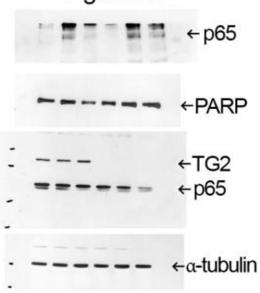

Supplementary Figure 8

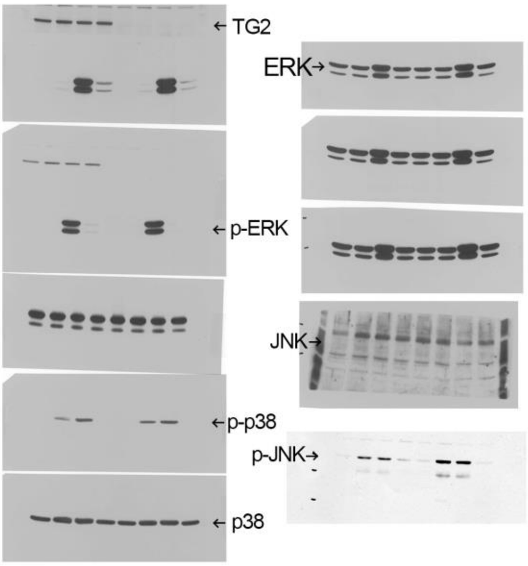

Supplementary Figure 10

Legends to Supplementary Figures

Supplementary Figure 1 Effect of TG2 siRNA #2 on osteoclast differentiation. **(a)** BMMs were transfected with control or TG2 siRNA #2 for 18 h. The efficiency of knockdown was determined by real-time PCR. **(b)** BMMs transfected with control or TG2 siRNA #2 were cultured with M-CSF plus RANKL for three days. Cells were stained for TRAP activity. Scale bar, 200 μm. **(c)** TRAP-positive MNCs (≥3 nuclei) were counted as osteoclasts. The surface area per osteoclast was measured using the Osteomeasure program. $^{**}P < 0.005$ versus control siRNA.

Supplementary Figure 2 Effect of TG2 knockdown on osteoclastic differentiation from RAW264.7 cells. **(a)** RAW264.7 cells were transfected with control or TG2 siRNA for 18 h. The efficiency of knockdown was determined by real-time PCR. **(b)** RAW264.7 cells transfected with control or TG2 siRNA were cultured with RANKL for three days. Cells were stained for TRAP activity. Scale bar, 200 μm. **(c)** TRAP-positive MNCs (≥3 nuclei) were counted as osteoclasts. The surface area per osteoclast was measured using the Osteomeasure program. $^{**}P < 0.005$ versus control siRNA.

Supplementary Figure 3 Effect of TG2 knockdown in pOCs on osteoclast differentiation. **(a,b)** BMMs were cultured with M-CSF plus RANKL for a day and cells were transfected with control or TG2 siRNA. Cells were then cultured with M-CSF and RANKL for 3 days before staining for TRAP activity. Scale bar, 200 μm. TRAP-positive MNCs (≥3 nuclei) were counted as osteoclasts. The surface area per osteoclast was measured using the Osteomeasure program. **(c-e)** BMMs cultured with M-CSF plus RANKL for two days were transfected with control or TG2 siRNA. After further culturing for 2 days with M-CSF and RANKL, cells were stained for TRAP activity. Scale bar, 200 μm. **(d)** TRAP-positive MNCs (≥3 nuclei) were counted as osteoclasts. The surface area per

osteoclast was measured using the Osteomeasure program. **(e)** The mRNA levels of *TRAP* and *DC-STAMP* were measured by real-time PCR. $^{**}P < 0.005$ versus control siRNA.

Supplementary Figure 4 Effects of TG2 knockdown on MAPK signaling pathways. BMMs were transfected with control or TG2 siRNA. At 48 hr after siRNA transfection, BMMs were deprived of serum and factors for 5 h and re-stimulated with RANKL (500 ng/ml) for the indicated times. Whole cell lysates were subjected to Western blot analysis with indicated MAPKs antibodies. Full length Western blots are presented in Supplementary Figure 10.

Supplementary Figure 5 TG2-deficient osteoclasts show enhanced sealing zone formation. BMMs obtained from WT and *TG2* knockout mice were cultured with M-CSF and RANKL for 3 days. Cells were stained with rhodamine-phalloidin for F-actin and DAPI for nuclei. Scale bar, 100 μm. Sealing zone (SZ) area per osteoclast and number of actin rings were measured. $^{**}P < 0.005$ versus WT cells.

Supplementary Figure 6 Cell proliferation of *TG2* knockout BMMs. BMMs from WT and *TG2* knockout mice were cultured for the indicated days and CCK assay was performed.

Supplementary Figure 7 Nuclear NFATc1 in *TG2* KO cells. BMMs were treated with M-CSF and RANKL for two days. Cells were stained for NFATc1 and laminB. Anti-laminB staining marked the nuclear envelope. Cells with nuclear NFATc1 staining were counted. Scale bar, 20 μm.

Supplementary Figure 8 MAPKs signaling pathways in TG2 knockout BMMs. WT and *TG2* knockout BMMs were deprived of serum and factors for 5 h and re-stimulated with RANKL (500

ng/ml) for the indicated times. Whole cell lysates were analyzed by Western blot using MAPKs antibodies. Full length Western blots are presented in Supplementary Figure 10.

Supplementary Figure 9 Full length gels and Western blots from Figures 1 and 3. Labels beside the Western blot indicate the antibody used.

Supplementary Figure 10 Full length Western blots from Figures 4, 5, 7, 8, Supplementary Figures 4 and 8. Labels beside the blot indicate the antibody used.

Table 1 Primers for chromatin immunoprecipitation assay.

**Binding sites**	**Positions**	**Primer sequence**	**Product size (bp)**
1	-1107	Sense: GGGAGAGATGATTTTTAAAAATG	119
		Antisense: GCCAGCATCCCATCACAG	
2	-979	Sense: CTGTGAATTGGAGGATCCCTGCTG	99
		Antisense: GCGTGGACCTTGCATTCCTGCTTC	
3	-846	Sense: GCCATCATCACAGGATGTCCTTCCTTC	121
		Antisense: GGGGCAGTGAGTGGAAAGCTGTTGGAG	
4	-387	Sense: CGAAGTACGTCGGATCCTGTAATTG	120
		Antisense: GTCCTCCGGATCGCTAGCTTCCTG	
5	-373	Sense: GGAGGAAGTTAGGTCTACCTAAGCTG	91
		Antisense: CTAAGGCGGTTCTCCTCTAGTATTAAAC	
6	-255	Sense: CGTAGTGTGGGTAAACATGGAG	106
		Antisense: GCGACCCGCGGCAGCTTCCTCTG	
7	-188	Sense: AAGCTGCCGCGGGTCGCAGTC	118
		Antisense: GACGGTCTGATTCACTCCTACCAG	

*Primer sequences were obtained from Wang X, Ma K, Chen M, Ko K-H, Zheng BJ, Lu L (2016) IL-17A Promotes Pulmonary B-1a Cell Differentiation via Induction of Blimp-1 Expression during Influenza Virus Infection. PLoS Pathog 12(1):e1005367. doi:10.1371/journal.ppat.1005367.

Table 2 Primers for RT-PCR and quantitative real-time PCR experiments.

Gene		Sequence	Size (bp)	GenBank accession #
(TG family)		5'- -3'		
mouse *TG1*	Sense	CATTGGCACACTCATTGTCA	143	NM_001161715
	Antisense	ACACATTAGGTTTGCTGCCA		
mouse *TG2*	Sense	TATGATGCACCCTTCGTGTT	135	NM_009373
	Antisense	GCCCACACTCTTAGTGCTGA		
mouse *TG3*	Sense	CCATTGGCAAATACATCAGC	148	NM_009374
	Antisense	CCGAAAGATGCGTTAGGTTT		
mouse *TG4*	Sense	TTGTGTTCACGGAGGTCAAT	150	NM_177911
	Antisense	TATCTTCACGCCTGTTCTCG		
mouse *TG5*	Sense	GGTGTTCCTAAAGGCTCTGC	142	NM_028799
	Antisense	ATCACTGGGTTGAAGGGAAG		
mouse *TG6*	Sense	CAGGTGCATCAGTACCAAGG	138	NM_001289747
	Antisense	CAGGCTTCCACACTTAGCAG		
mouse *TG7*	Sense	CCAATTTCCATTCTGCACAC	136	NM_001160424
	Antisense	ATCATCCAGCACTCATTCCA		
mouse *Factor XIII A*	Sense	TGAGCTCCAAACTCACCAAG	145	NM_028784
	Antisense	CGGTACATGCCATCACTGTT		
(OC makers)		5'- -3'		
mouse *c-Fos*	Sense	ACTTCTTGTTTCCGGC	233	NM_010234
	Antisense	AGCTTCAGGGTAGGTG		
mouse *NFATc1*	Sense	CCAGTATACCAGCTCTGCCA	188	BC061509
`	Antisense	GTGGGAAGTCAGAAGTGGGT		
mouse *TRAP*	Sense	CGACCATTGTTAGCCACATACG	77	BC019160
	Antisense	TCGTCCTGAAGATACTGCAGGTT		
mouse *DC-STAMP*	Sense	GGGTGCTGTTTGCCGCTG	132	NM_029422
	Antisense	CGACTCCTTGGGTTCCTTGCT		
mouse *Atp6v0d2*	Sense	AGTGCAGTGTGAGACCTTGG	133	NM_175406
	Antisense	TCTGCAGAGCTTCTTCCTCA		
mouse *Blimp1*	Sense	GCCCAGTGTCACAAGAGCTA	141	NM_007548
	Antisense	GCTGATGTCGAACCTCTCAA		
(control)		5'- -3'		
mouse *HPRT*	Sense	GTGATTAGCGATGATGAACCA	149	NM_013556
	Antisense	CCCATCTCCTTCATGACATCT		
